# Formation of a Family of Long Intergenic Noncoding RNA Genes with an Embedded Translocation Breakpoint Motif in Human Chromosomal Low Copy Repeats of 22q11.2—Some Surprises and Questions

**DOI:** 10.3390/ncrna4030016

**Published:** 2018-07-20

**Authors:** Nicholas Delihas

**Affiliations:** Department of Molecular Genetics and Microbiology, School of Medicine, Stony Brook University, Stony Brook, New York, NY 11794-5222, USA; Nicholas.delihas@stonybrook.edu; Tel.: +1-631-286-9427

**Keywords:** formation of lincRNA genes, chromosomal low copy repeats, segmental duplications, 22q11.2, translocation breakpoint sequence, directed mutations, palindromic AT-repeats, human satellite 1, HSAT I, formation of RNA exons

## Abstract

A family of long intergenic noncoding RNA (lincRNA) genes, *FAM230* is formed via gene sequence duplication, specifically in human chromosomal low copy repeats (LCR) or segmental duplications. This is the first group of lincRNA genes known to be formed by segmental duplications and is consistent with current views of evolution and the creation of new genes via DNA low copy repeats. It appears to be an efficient way to form multiple lincRNA genes. But as these genes are in a critical chromosomal region with respect to the incidence of abnormal translocations and resulting genetic abnormalities, the 22q11.2 region, and also carry a translocation breakpoint motif, several intriguing questions arise concerning the presence and function of the translocation breakpoint sequence in RNA genes situated in LCR22s.

As thousands of long noncoding RNA (lncRNA) genes have recently been detected [1,2,3,4], one of the interesting problems is their formation. These RNA genes are highly diverse and several different pathways concerning their origins have been outlined [5,6,7,8,9,10,11,12]. Here we analyze and discuss the formation of a family of long intergenic noncoding RNA (lincRNA) genes via gene sequence duplication [13]. We concentrate on duplications that occurred specifically in chromosomal low copy repeats (LCR22) in chromosome 22 (chr22) [14,15,16] that are in or close to the 22q11.2 chromosomal region. These duplications evolved into eight lincRNA genes that form part of the *FAM230* lncRNA gene family [13] (see also Table S1 for the characteristics of these genes). Although LCR22s provide the means for formation of multiple lincRNA genes, 22q11.2 is a critical chromosomal region, prone to deletions that are mediated by LCR22s and result in genetic disorders such as DiGeorge Syndrome and velo-cardio-facial syndrome [14,15,17]. In addition, the DNA translocation type A breakpoint motif (TBTA), which is directly involved in 22q11.2 deletions [18,19], is incorporated in newly formed lincRNA genes in LCR22s. Significantly, the TBTA sequence present in these genes is modified by highly selective deletion mutations. This offers intriguing questions but presents a possible paradox.

Fifteen lincRNA genes originated by duplication of the sequence of lincRNA gene *FAM230C*, which is situated in chromosome 13 (chr13) [13]. The *FAM230C* gene sequence is the source of formation of two primary groups of genes, where the group category depends on whether the gene originated from the 5′ half or 3′ half of *FAM230C* sequence. Eight lincRNA genes originated from copies of the 3′ end sequence of *FAM230C* and were formed specifically in LCR22s in chr22. These genes include the TBTA motif originally derived from copies of the *FAM230C* gene (Figure 1). Translocation breakpoint type A motif and its related translocation breakpoint sequences have been shown to undergo DNA stand breakage via palindromic hot spot stem loop structures and cruciforms leading to chromosomal translocation [19,20,21].

An exception is a ninth gene, *AP000552.3* ENSG00000237407, which is a separate type—a small gene that is antisense to *AP000552.1*. ENSG00000206142 (one of the eight genes), and it does not harbor the TBTA. The eight lincRNA genes all display a tissue specificity of RNA transcript expression with major expression only in the testes [13,22]; however, RNA transcript functions are not known.

On the other hand, a heterogeneous group of six genes was formed from the 5′ half of the *FAM230C* gene sequence and none contain the TBTA motif. These genes reside in chromosomes other than chr22 (Figure 1). In addition, RNA expression from these genes is varied, with some such as *DUXAP9* that shows RNA expression in multiple tissues (http://useast.ensembl.org/Homo_sapiens/Gene/ExpressionAtlas?db=core;g=ENSG00000225210;r=14:19062316-19131167) [23]. Thus there appear to be two very different categories of genes formed from the lincRNA *FAM230C* gene sequence, whereby cellular regulatory processes determine where the genes are formed and what sequences they contain.

Multiple copies of the *FAM230C* sequence in LCR22s are the result of a large expansion of the sequence involving DNA segmental duplications, with the subsequent formation of the eight lincRNA genes. Although only the 3′ half sequence of *FAM230C* is used for gene formation, remnants of the 5′ half sequence of *FAM230C* are present in LCR22s and these are not part of the new RNA genes [13].

Thus, LCR22s are a vehicle for creation of multiple lincRNA genes. This is in keeping with the concept that LCRs, or segmental duplications are a major force in human evolution and formation of new genes [24,25,26,27,28]. Genes formed from copies of the 5′ half of *FAM230C* (Figure 1) do not appear to involve segmental duplications and for the most part, these are single genes formed in different chromosomes.

Another aspect of this process is more difficult to understand. The *FAM230C* sequence carries the TBTA motif and *FAM230C* sequence duplications spread multiple copies of the TBTA motif in LCR22s. The TBTA motif is sequestered within RNA genes that are formed in LCR22A, B, D and F [13] (Figure 1). These LCR22s are close to each other and less than 10 megabase-pairs apart, in or near the 22q11.2 region [29]. Low copy repeats closer than 10 megabase-pairs are prone to misalignment with resultant chromosomal deletions or duplications [30]. LCR22s are known to participate in meiotic nonallelic homologous recombinations that lead to 22q11.2 deletions and subsequent genetic diseases [31]. The 22q11.2 region displays the most common chromosomal microdeletion genetic disorder “estimated to result mainly from de novo nonhomologous meiotic recombination events occurring in approximately 1 in every 1000 fetuses” [32]. It is also estimated that ~1 in 3000 to 4000 infants are born with the 22q11.2 deletion [33]. Thus, the 22q11.2 region is associated with a significant incidence of genetic abnormalities that involve participation by LCR22s. 

At the molecular level, the TBTA and its related repeat sequences contain palindromic AT-rich repeat sequences (PATRR) that form a very long stem loop (Figure 2). These have loop breakpoint sites directly associated with translocations that can result in genetic disorders involving 22q11.2 [18,19,34,35]. Specifically, PATRR breakpoint sites have been found in LCR22B [18,19,36]. This raises the question of the presence of the TBTA motif in lincRNA genes situated in LCR22s.

Of major significance, TBTA sequences in the eight lincRNA genes, including the *FAM2230C*, have a 5′ end segment of the PATRR stem loop deleted. As an example, Figure 3 shows the deletion in one of the RNA genes, *LINC01660*; the green color highlights the missing nucleotide sequence. This deletion totally disrupts the PATRR secondary structure and the resultant unfolded structure is unlikely to produce strand breakage or a translocation site. As the deletion is also present in the *FAM230C* TBTA sequence, F*AM230C* duplications in LCR22s may have passed on the deletion to all the eight genes during their formation. The PATRR disruption could be to insure that there are no PATRR-related translocation breakpoint sites that may stem from the eight RNA genes in LCR22s in 22q11.2.

In addition to the PATRR, the TBTA has another section that can form a long stem loop, the AT-rich region #2 (Figure 2). Tong et al. [39] showed that AT-rich region #2 present in a translocation breakpoint element that is related to the TBTA, displays translocation activity, albeit representing a rare translocation event and shows a low frequency of translocation (1.52 × 10^−7^) as opposed to the TBTA PATRR (ID: AB261997.1), which has a 10^−4^–10^−5^ frequency of translocation [19]. Analysis of TBTA sequences from the eight lincRNA genes shows deletions of the AT-rich region #2, but surprisingly, only in two of the eight lincRNA genes, *LINC01658* and *LINC01662.* As an example, Figure 4 shows the AT-rich region #2 and the PATRR-associated AT-rich sequences totally deleted in *LINC01658*. The sequences between the arrows in Figure 4 denote deleted areas. In addition, a complete elimination of AT-rich sequences occurred in this gene with an additional deletion, that of the smaller AT-rich region #1, with the exception of positions 360–366 (Figure 4). Essentially, *LINC01658* is devoid of AT-rich sequences. In contrast, an example of the presence of the entire AT-rich #2 motif is in *LINC01663*, one of the six RNA genes that have the AT-rich region #2 (Figure S1). As expected, the AT sequences are highly variable. There is also a very large number of AT bases present in *LINC01663* relative to the TBTA AT-rich region (Figure S1), indicating a robust expansion of AT sequences in this gene. This is in sharp contrast to *LINC01658* that is devoid of AT-rich sequences.

This seemingly is paradoxical, as one would expect AT-rich #2 sequences to be deleted in all lincRNA genes to eliminate the possibility of a translocation breakpoint sequence evolving in RNA genes present in LCR22s. AT-rich sequences are highly unstable, undergo extensive point mutations, insertions, deletions, and readily form long stem-loop secondary structures. For example, random DNA sequences with 500 bases containing 95% A + T can generate 50–100 base pair stems of a stem loop structure. None of the lincRNA genes display very long, perfect AT stem loops, but one cannot rule out potential breakpoint sequences evolving from AT-rich variable sequences in one or more lincRNA genes. Does the cell tolerate a probability of a rare translocation event occurring within RNA genes in LCR22s that carry AT-rich #2 sequences? This is in a background of the 22q11.2 region that already is highly problematic in terms of incidence of genetic disease stemming from abnormal translocations.

Why does the *FAM230C* gene, which is in chr13, harbor the TBTA? And why is the translocation breakpoint motif specifically added to newly formed RNA genes in LRC22s? We do not have enough information to answer or comment on the first question, but in terms of the second, the TBTA carries the human satellite 1, HSAT I (Figure 4) [13,21,40]. Segments of the HSAT I sequence form the entire exon1 of several annotated RNA transcripts from lincRNA genes [41] (see Figure S2). Thus, the TBTA sequence helps form lincRNA gene structure by carrying the HSAT I satellite, and this demonstrates a role for a satellite sequence in development of lincRNA gene and transcript exon sequence. This may be a secondary or separate role of the TBTA and does not address the addition of the motif to the RNA genes that are specifically in LCR22s. Protein genes are known to carry breakpoint sequences; about 2000 genes have been detected that harbor purine/pyrimidine tracts that form long stem loops [42]. Perhaps genes are storage and protection sites for these elements.

The TBTA and its related motifs are present in nonhuman primates [21,43]. In addition, the 3’ half of the *FAM230C* sequence is found in chr22 of the chimpanzee genome with a sequence identity of 97% compared with the human *FAM230C* [41] (Figure S3). It appears to be an ancient and highly conserved sequence present in a common ancestor of humans and chimpanzees. However, neither the entire *FAM230C* sequence or the eight related gene sequences have been detected as complete sequences, or as yet have been annotated in the chimpanzee or other primates. There must be more complete genomic sequences from the chimpanzee and other primates, as well as better lincRNA gene annotations to determine if these genes are present in the chimpanzee or other primate genomes, or if they are specific to humans.

We also do not know the evolutionary relationship between *FAM230C* and the putative protein gene *FAM230A* (Ensembl: ENSG00000277870) in human chr22. The *FAM230A* gene is only partially defined as there is a remaining 50,000 bp unsequenced gap in the central portion of the gene.

It is surprising that deletion mutations eliminated the PATRR structure from the eight lincRNA genes in LCR22s, yet six genes are left with AT-rich sequences, some having an excess of AT residues that we hypothesize may potentially evolve into breakpoint structures. 22q11.2 is a complex region. We have some, but not a full understanding of the relationship of these lincRNA genes to the region, to the translocation breakpoint motif, and of RNA transcript function. What does appear obvious is the high specificity in types of mutations that occurred in the RNA genes, the regulation in chromosomal placement of genes, the mechanism of gene formation, and the importance of a DNA satellite in RNA exon sequence formation. However, to our knowledge, the eight genes of the *FAM230* lincRNA family are the first lincRNA genes known to be formed by DNA segmental duplications. As segmental duplications are considered a major factor in human evolution and creation of new genes [24,25,26,27,28], there may be other lincRNA gene families formed by this pathway.

## Figures and Tables

**Figure 1:**
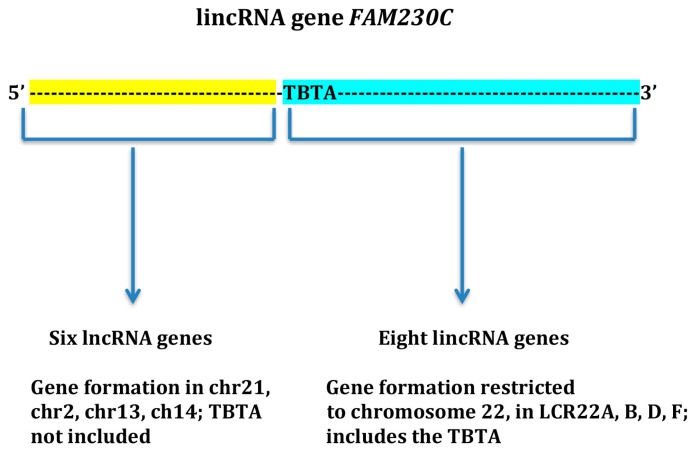
A schematic of 5′ and 3′ sections of the long intergenic noncoding RNA (lincRNA) gene *FAM230C* present in chr13 that form two distinct groups of long noncoding RNA (lncRNA) genes. Based on reference [13]. Abbreviations: chr, chromosome; TBTA, translocation breakpoint type A; LCR22, low copy repeats in chr22.

**Figure 2:**
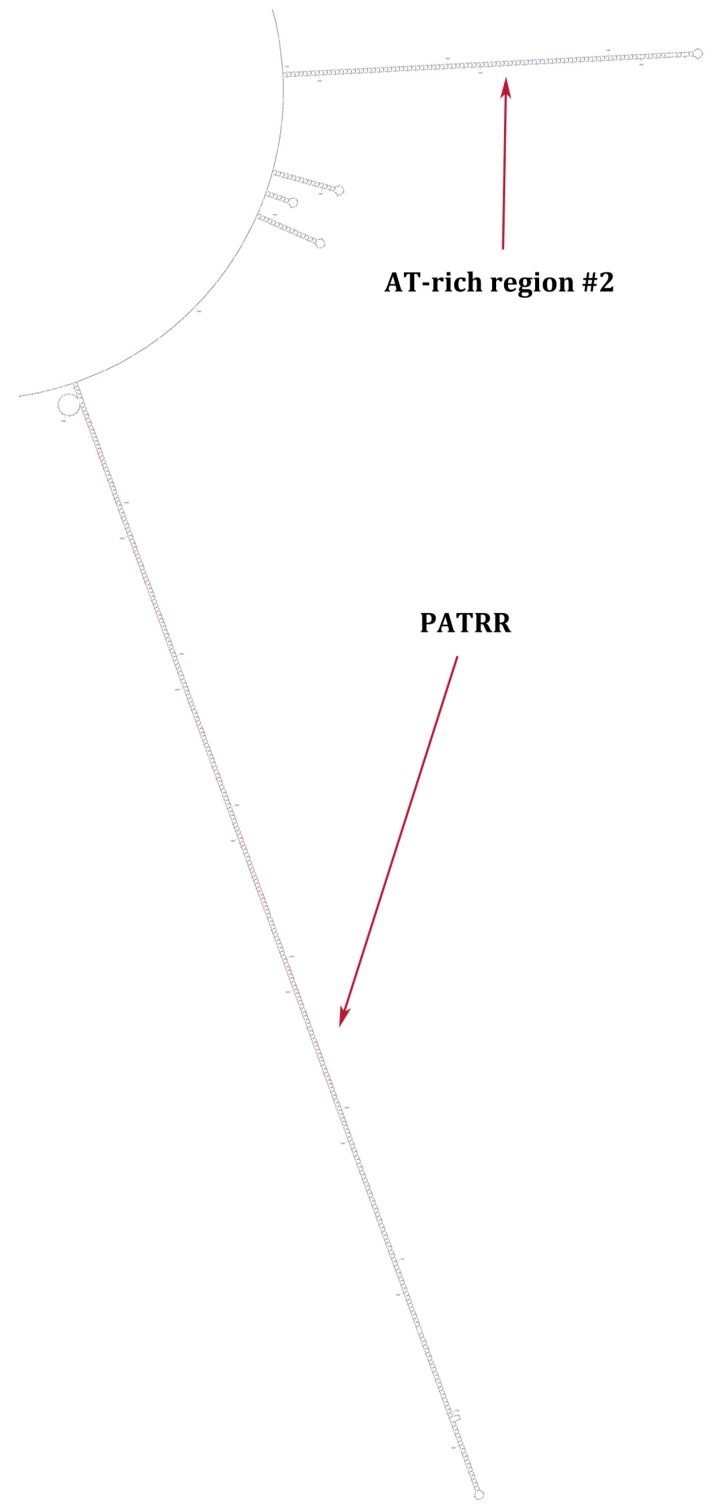
A segment of the secondary structural model of the TBTA (GenBank sequence ID: AB261997.1) showing two long stem loops: the 294 bp palindromic AT-rich repeat sequences (PATRR) and the 104 bp stem loop formed by AT sequences from AT-rich region #2. The PATRR has a significant number of G:C bonds in the lower portion of the stem but is A:T base pair rich in the upper portion close to the loop side. The AT-rich region #2 stem loop consists entirely of A:T bonds with the exception of two G:T bonds. The Zuker DNA folding program [37] was used to generate the secondary structure.

**Figure 3:**
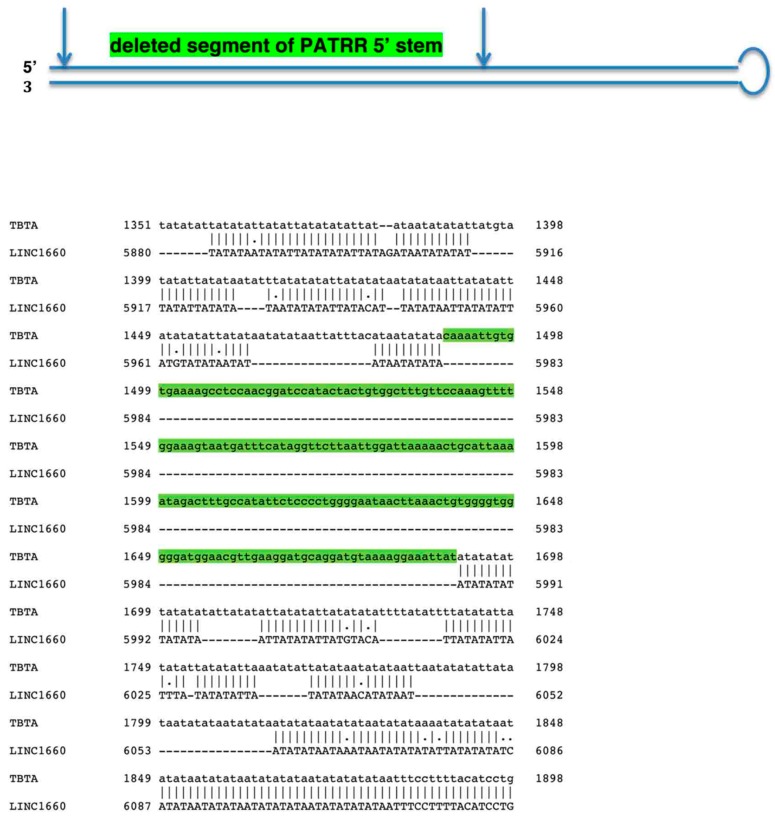
(**Top**) Schematic of PATRR stem loop. The section between arrows denote sequences from the 5’ half missing in gene *LINC01660.* (**Bottom**) Alignment of the nucleotide sequences of the TBTA and L*INC01660*. The alignment was determined by the Emboss Needle Pairwise Sequence Alignment program (https://www.ebi.ac.uk/Tools/psa/emboss_needle/nucleotide.html) [38]. Green color highlights the TBTA nucleotide sequence missing in *LINC01660* equivalent to TBTA nucleotide positions 1489–1690. This sequence has a large number of G and C residues that form a number of G:C pairs at the base of the 5′ side of the double stranded stem of the PATRR that stabilizes the stem. The G:C pairs as well as other base pairs are missing in *LINC01660* that has the PATRR 5’ section deleted.

**Figure 4:**
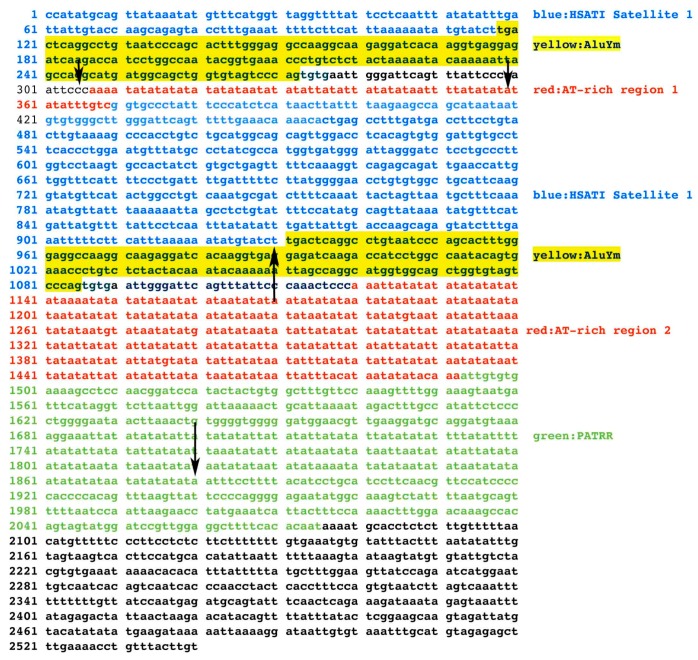
The TBTA sequence, highlighting the various elements it harbors, including the PATRR and AT-rich region #2 and AT-rich region #1. The sequence is from National Center of Biotechnology information (NCBI) GenBank: AB261997.1. The figure is modified from reference [13]. Two arrows (bottom) delineate the regions deleted in the RNA gene *LINC01658* sequence that is equivalent to TBTA positions 930–1880, and encompasses the AluYm transposable element (in yellow), the entire AT-rich region #2 (in red), the 5’ side of the PATRR and includes all of the PATRR-associated AT-rich sequences up to position 1880 (in green). The AT-rich region #1 is also deleted (delineated by arrows at positions 305 and 359) with the exception of the very small AT sequence, positions 360–366. These deletions essentially result in RNA gene *LINC01658* having no AT-rich sequences.

## Supplementary Materials

The following are available online at http://www.mdpi.com/2311-553X/4/3/16/s1. Table S1: Properties of lincRNA genes in chr22 (from reference [13]), Figure S1: Nucleotide sequence alignment of the TBTA AT-rich region #2 with the AT-rich region of *LINC01663*, Figure S2: Nucleotide sequence alignment of the exon 1 sequence from RNA transcript *LINC01660*-203 with the TBTA, Figure S3: Nucleotide sequence alignment of the RNA gene *FAM230C* with a segment of chr22 from the chimpanzee genome.
