# Supplementary material for: Formation of a Family of Long Intergenic Noncoding RNA Genes with an Embedded Translocation Breakpoint Motif in Human Chromosomal Low Copy Repeats of 22q11.2—Some Surprises and Questions
Source: Noncoding RNA. 2018 Jul 20;4(3):16. doi: 10.3390/ncrna4030016 (PMC6162681; doi:10.3390/ncrna4030016)
Supplement: Supplementary file 1 [file ncrna-04-00016-s001.zip › Figure S1.pdf]

Formation of a family of long intergenic non-coding RNA genes with an embedded translocation breakpoint motif in human chromosomal Low Copy Repeats of 22q11.2 - some surprises and questions

Nicholas Delihas
Department of Molecular Genetics and Microbiology
School of Medicine
Stony Brook University
Stony Brook, New York, 11794-5222 USA

Supplementary File 2

```
TBTA            1105 ---tattcc---------------caaactccc----------aaattat   1126
                        .|||||               .|||.||.|          |||||||
LINC01663       2600 TTGCATTCCAGCCTGTGCAACAGAAAAAGTCTCTGTCTCAAAAAAATTAT   2649

TBTA            1127 atatatat-atatatataaaatata---------------------tata   1154
                     |||||||| ||||||||.|.|||||                     ||||
LINC01663       2650 ATATATATAATATATATTATATATATTTTATATAACATATATTTTTTATA   2699

TBTA            1155 taatatataatatataatatatataatatatataatata-----------   1193
                     |.||||||||||||||.|||||||.||||||||.|||||
LINC01663       2700 TTATATATAATATATATTATATATTATATATATTATATATTATATATTAT   2749

TBTA            1194 --------------------------------------------------   1193

LINC01663       2750 ATAAAATGTATTATATATTATATATTATATCTATTATATACATTATATAT   2799

TBTA            1194 ------------------------------------------taatatat   1201
                                                               ||||||||
LINC01663       2800 AGTATATATTATATACCATATATAATATATGTAGTATACATGTAATATAT   2849

TBTA            1202 aatatatattatat------------------------------------   1215
                     ||||||||||||||
LINC01663       2850 AATATATATTATATGTCATATCATATATAATGTCATATTATATATCATAT   2899

TBTA            1216 --------------atataatatataatatata-----------------   1234
                                   |||||||||||||||||||
LINC01663       2900 ATAATATATATTACATATAATATATAATATATATTACATATAATATATAA   2949

TBTA            1235 -atatatt-------atatgtaatatatatta-----aatatataat---   1268
                      |||||||       ||||.||||||||||||     ||||||||||
LINC01663       2950 TATATATTACATATAATATATAATATATATTACATATAATATATAATACA   2999

TBTA            1269 ------gtataatatatgatatataatatat-------------------   1293
                           .||||||||||.|||||||||||||
LINC01663       3000 TATTACATATAATATATAATATATAATATATATTACATATAATATATAAT   3049

TBTA            1294 ----------------------tatatattatatattat-----------   1310
                                           |||||||||||||||||
LINC01663       3050 ATATAATATATATTACATATTATATATATTATATATTATACATTATATAT   3099

TBTA            1311 ---------------atatataat--------------------------   1319
                                    |||||||||
LINC01663       3100 ATTACATCATATATCATATATAATATCTTATATCATATATTATATTATAT   3149

TBTA            1320 -------------atatattatatatta--------tatattatatatat   1348
                                  |||||||||||||||        |||||||||||||.
LINC01663       3150 ATAATGTATCATAATATATTATATATTATCATATATTATATTATATATAA   3199

TBTA            1349 tatat------atattatatattatattatatatat-----tatataata   1387
                     |||||      ||||.||||||||||||||||||||     |||||.|||
LINC01663       3200 TATATCATAAGATATAATATATTATATTATATATATCATAATATATTATA   3249

TBTA            1388 ---tatattatgtatatattatataatat------ttata-tatatta--   1425
                        |||||||| ||.||||||||||||||      ||||| |||||||
LINC01663       3250 TATTATATTAT-TACATATTATATAATATTACATATTATATTATATTATT   3298

TBTA            1426 --tatataatatataa----ttat------------atattatatatatt   1457
                       ||||||.|||.|||    ||||            ||||||||||||||
LINC01663       3299 ATTATATATTATGTAATATTTTATATTATATATGTAATATTATATATATT   3348

TBTA            1458 ata---taatatataattat----ttacataatatatacaaaattgtgtg   1500
                     |||   |||||||| |||||    |.|||||.||||||.||.|||.|.|
LINC01663       3349 ATATTGTAATATAT-ATTATAGTATGACATATTATATATAATATTATAT-   3396
```

Figure S1. Nucleotide sequence alignment of the TBTA AT-rich region #2 with the AT-rich region of LINC01663 that is highlighted in red letters. TBTA position 1120 is the start of the AT-rich region #2 and position 1492 is the end. The position 1493 is the start of the PATRR sequence in the TBTA. The EMBL-EBI program Clustal Omega was used (https://www.ebi.ac.uk/Tools/msa/clustalo/)
