# Supplementary material for: Formation of a Family of Long Intergenic Noncoding RNA Genes with an Embedded Translocation Breakpoint Motif in Human Chromosomal Low Copy Repeats of 22q11.2—Some Surprises and Questions
Source: Noncoding RNA. 2018 Jul 20;4(3):16. doi: 10.3390/ncrna4030016 (PMC6162681; doi:10.3390/ncrna4030016)
Supplement: Supplementary file 1 [file ncrna-04-00016-s001.zip › Figure S2.pdf]

Formation of a family of long intergenic non-coding RNA genes with an embedded translocation breakpoint motif in human chromosomal Low Copy Repeats of 22q11.2 - some surprises and questions

Nicholas Delihas
Department of Molecular Genetics and Microbiology
School of Medicine
Stony Brook University
Stony Brook, New York, 11794-5222 USA

Supplementary File 3

Annotated transcript: LINC01660-203

Transcript ID: ENST00000618517.4

Transcript Support Level (TSL) is 2

>exon1.LINC01660-203
GATTAGGGATCTCCTGCCCTTGGTCGTAAGTGCCACTACCTGTGCTGAGTTTTTCAAAGG
TCAGAGCAGATTGAACCATTGTGGTTTCATTTTCCCTGATTTTGATTTTTCTTATGGGGA
ACCTGTGTGGCTGCATTCAAG

Query: TBTA
Subject: LINC01660-203.exon1

Score	Expect	Identities	Gaps	Strand
250 bits(135)	1e-70	139/141(99%)	0/141(0%)	Plus/Plus

```
Query  580   GATTAGGGATCTCCTGCCCTTGGTCCTAAGTGCCACTATCTGTGCTGAGTTTTTCAAAGG  639
             ||||||||||||||||||||||||| |||||||||||| |||||||||||||||||||||
Sbjct  1     GATTAGGGATCTCCTGCCCTTGGTCGTAAGTGCCACTACCTGTGCTGAGTTTTTCAAAGG  60

Query  640   TCAGAGCAGATTGAACCATTGTGGTTTCATTTTCCCTGATTTTGATTTTTCTTATGGGGA  699
             ||||||||||||||||||||||||||||||||||||||||||||||||||||||||||||
Sbjct  61    TCAGAGCAGATTGAACCATTGTGGTTTCATTTTCCCTGATTTTGATTTTTCTTATGGGGA  120

Query  700   ACCTGTGTGGCTGCATTCAAG  720
             |||||||||||||||||||||
Sbjct  121   ACCTGTGTGGCTGCATTCAAG  141
```

Figure S2. Nucleotide sequence alignment of the exon1 sequence from RNA transcript LINC01660-203 (subject) with the TBTA (query). TBTA positions 580-720 are part of the HSATI sequence (see Figure 4, text).
