# Supplementary material for: Formation of a Family of Long Intergenic Noncoding RNA Genes with an Embedded Translocation Breakpoint Motif in Human Chromosomal Low Copy Repeats of 22q11.2—Some Surprises and Questions
Source: Noncoding RNA. 2018 Jul 20;4(3):16. doi: 10.3390/ncrna4030016 (PMC6162681; doi:10.3390/ncrna4030016)
Supplement: Supplementary file 1 [file ncrna-04-00016-s001.zip › Figure S3.pdf]

Formation of a family of long intergenic non-coding RNA genes with an embedded translocation breakpoint motif in human chromosomal Low Copy Repeats of 22q11.2 - some surprises and questions

Nicholas Delihas
Department of Molecular Genetics and Microbiology
School of Medicine
Stony Brook University
Stony Brook, New York, 11794-5222 USA

Supplemental File 4 Figure S3

Nucleotide sequence alignment

Query: FAM230C gene sequence

Subject: DNA sequence from GenBank: AC191381.3. DNA obtained from white blood cells of male chimpanzee (Pan troglodytes, 'Clint', Yerkes #C0471 as described, GenBank: AC191381.3).

Pan troglodytes BAC clone CH251-588A5 from chromosome 22, complete sequence
Sequence ID: AC191381.3 **Length:** 226324

Alignment

Score	Expect	Identities	Gaps	Strand
28513 bits(15440)	0.0	16622/17181(**97%**)	127/17181(0%)	Plus/Minus

```
Query  20796   AAATtatatatatatgtat-tttata-tatataattgtatatataattatataatata  20853
               ||||||||||| || || |||  ||||| ||||| ||  ||||||| || ||||| ||||
Sbjct  193653  AAATTATATATTTA-AT-TATAATTATATTATAT-ATAATATATATTAT-ATATATTATA  193598

Query  20854   taatatagatatataatatatataatatattatttatatat-t-atatatattgtata-a  20910
               || |||| | || || ||||||| ||| || | |||||||| | |||| |||| |||| |
Sbjct  193597  TA-TATA-AAATGTATTATATATTATACAT-A-TTATATATGTAATATGTATTATATATA  193542

Query  20911   -tatatattatacatatataattatat-ttataatatatgtataATTTCCTTTTACATCC  20968
                ||||||| ||  ||||||| |||| |  |||||||||| ||||||||||||||||||||
Sbjct  193541  TTATATATAATGTATATATA-TTATGTAATATAATATATATATAATTTCCTTTTACATCC  193483

Query  20969   TGCATCCTTCAACGTTCCATCCCCCACCCCACAGATTAA-TTATTCCCTAGGGGAGAATA  21027
               ||||||||||||||||||||||  ||||||||||||||| |||||||||||||||||  |
Sbjct  193482  TGCATCCTTCAACGTTCCATCCGTCACCCCACAGATTAAGTTATTCCCTAGGGGAGAGGA  193423

Query  21028   TGGCAAAGTCTATTTTAATTCAGTTTTT-AACCTAATTAAGAACCTATGAAATCATTACT  21086
               ||||||||||||||||||| |||||||| |||||||||||| | ||||||||||||||||
Sbjct  193422  TGGCAAAGTCTATTTTAATGCAGTTTTTTAACCTAATTAAGGAACTATGAAATCATTACT  193363

Query  21087   TTCCAAAACTTTGGAACAAAGCCACAGTAGTATGGATGGGTTGGAGGCTTTTCACACCAT  21146
               |||||||  |||| |||||||||||||||||||| |||||||||||||||||||||| ||
Sbjct  193362  TTCCAAATATTTGAAACAAAGCCACAGTAGTATGCATGGGTTGGAGGCTTTTCACACAAT  193303

Query  21147   AAAATGTACCTATCTTTGTTTTTAACATGTTTTTCCCTTCCTCTCTTCtttttttGTGAA  21206
               ||||||||||||||||||||||||||||||||||||||||||||||||||||||||||||
Sbjct  193302  AAAATGTACCTATCTTTGTTTTTAACATGTTTTTCCCTTCCTCTCTTCTTTTTTTGTGAA  193243

Query  21207   ATGTGTATTTACTTTAATAAATTTGTAGTAAGTCATTTCCATTCACATATTAATTTTTA  21266
               |||||||||||||||||||||||||||||||||| |||||||||||||||||||||||||
Sbjct  193242  ATGTGTATTTACTTTAATAAATTTGTAGTAAGTCGTTTCCATTCACATATTAATTTTTA  193183

Query  21267   AAGTAATAAGAACGTGTATTGTCTGCGTGTGAAATAAAACTCACATTTATTTTTATGCTT  21326
               ||||||||| || ||||||||||| ||||||||||||||| ||| ||| |||||||||||
Sbjct  193182  AAGTAATAATAATGTGTATTGTCTACGTGTGAAATAAAACACAC-TTT-TTTTTATGCTT  193125

Query  21327   TTGGAGTTATCCAAAATCATGGAATTGTCAATCACAGTCAATCACCCAACCTACTCACCT  21386
               | ||||||||||||||||||||||||||| ||||||||||||||||||||||||||||||
Sbjct  193124  TGGGAGTTATCCAAAATCATGGAATTGTCCATCACAGTCAATCACCCAACCTACTCACCT  193065

Query  21387   TTCCAGTGTAATCTTAGTCAAAtttttttttGTTATCCAATGAGATGCAGTATTTCAACT  21446
               ||||||||||||||||||||||||||||||||||||||||||||||||||||||||||||
Sbjct  193064  TTCCAGTGTAATCTTAGTCAAATTTTTTTTTGTTATCCAATGAGATGCAGTATTTCAACT  193005

Query  21447   CAGAAAGATAAATAGAATTAATTGGTAGAGACTATTAACTAAGAACATACAGTTTTATTT  21506
               || ||||||||||||||| |||||||||||||||||||||||||||||||||||||||||
Sbjct  193004  CACAAAGATAAATAGAATGAATTGGTAGAGACTATTAACTAAGAACATACAGTTTTATTT  192945

Query  21507   ATACTCAGAAGCAAGTAGATTATGTACATATATATGAAGATTAAAATTAAAAGGATAATT  21566
               ||||||||||||||||||||||||||||||||||||||||| ||||||||||||||||||
Sbjct  192944  ATACTCAGAAGCAAGTAGATTATGTACATATATATGAAGATAAAAATTAAAAGGATAATT  192885

Query  21567   GTGTAAATTTGCCTGTAGAGAGCTTTGAAATCCTGTTTACTTGTTAATGCTGTTTTGATG  21626
               |||||||||||| ||||||||||||||||| |||||||||||||||||||||||||||||
Sbjct  192884  GTGTAAATTTGCATGTAGAGAGCTTTGAAAACCTGTTTACTTGTTAATGCTGTTTTGATG  192825

Query  21627   TATTGTGTGACTTTGTTCTCCCGACCCATCATCCAGAGCTCTCTGCAGGAGCTAAGTGCT  21686
               ||||||||||||| ||||||||||||||||||||||||||||||||||||||||||||||
Sbjct  192824  TATTGTGTGACTTAGTTCTCCCGACCCATCATCCAGAGCTCTCTGCAGGAGCTAAGTGCT  192765

Query  21687   CATCAGTTCCATGACTTGGCAACTGTCTAAGTTTAGAGGCACTTGTATTTGTTAGTAAAT  21746
               ||||||||||||||||||| ||||||||||||||||||||||||||||||||||||||||
Sbjct  192764  CATCAGTTCCATGACTTGGAAACTGTCTAAGTTTAGAGGCACTTGTATTTGTTAGTAAAT  192705

Query  21747   AAGGCAAGATGATATTGTTTCACAGGTTTTAGTGCAGAAGACTGAATAGATAAGCTGCTC  21806
               |||||||||||||||||||||||||| ||||||||||||||||||||||| |||||||||
Sbjct  192704  AAGGCAAGATGATATTGTTTCACAGGCTTTAGTGCAGAAGACTGAATAGAGAAGCTGCTC  192645

Query  21807   CACCCAGTACACTGGTGTTCATTTCATGGTCATCTCATCTGTTAACCATGGATAAAAAAC  21866
               |||||||||||||||||||||||||||||||||||||||||||| ||||||||| |||||
Sbjct  192644  CACCCAGTACACTGGTGTTCATTTCATGGTCATCTCATCTGTTAGCCATGGATATAAAAC  192585

Query  21867   ACTTATCTTCAATGATCTTCCTGTACATGTAAAAACACACCTGTCTACATGGCAGCAGTT  21926
               |||||||| |||||||||||||||||||||||||||||||||||||||||||||||||||
Sbjct  192584  ACTTATCTCCAATGATCTTCCTGTACATGTAAAAACACACCTGTCTACATGGCAGCAGTT  192525

Query  21927   GGACCTCACAACGTGGATTGTGCCTTCACCCTGGAATGTCTATGATGCCCTATCGACCAT  21986
               ||||||||||| ||||||||||||||||||||||||||||||||||||||||||||||||
Sbjct  192524  GGACCTCACAATGTGGATTGTGCCTTCACCCTGGAATGTCTATGATGCCCTATCGACCAT  192465

Query  21987   GGTGATGGGATTAGGGATCTCTTGCCCTTTGCTCTGCCACTGTCTGCACCAGCCAGGCCA  22046
               ||||||||||||||||||||||||||||||||||||||||||||||  ||||||||||||
Sbjct  192464  GGTGATGGGATTAGGGATCTCTTGCCCTTTGCTCTGCCACTGTCTGTGCCAGCCAGGCCA  192405

Query  22047   CTGGGCCATTGTGGCAGATGGTGATGCCCTTTGTGTGGAGCGCACTGTGTGTGCGCTGAG  22106
               ||||||||||||||| ||||||||||||||||||||||||||||||| ||||| ||||||
Sbjct  192404  CTGGGCCATTGTGGCGGATGGTGATGCCCTTTGTGTGGAGCGCACTGAGTGTGTGCTGAG  192345

Query  22107   GCAGACACAGTACTTTTGACAACTTTATCTGCTTCTTTCAAAACTGGGTCTGCATTATGA  22166
               |||||||||||||||||||||||||||||||||| |||||||||||||||||||||||||
Sbjct  192344  GCAGACACAGTACTTTTGACAACTTTATCTGCTTGTTTCAAAACTGGGTCTGCATTATGA  192285

Query  22167   TAAAGTGGTTGGTTTTAGGCCAAGCAGGTGCATGTCATAGAACAATGACTTGGGAGTTCA  22226
               |||||| ||||||||||||||||||||||| |||||||||||||||||||||||||||||
Sbjct  192284  TAAAGTTGTTGGTTTTAGGCCAAGCAGGTGTATGTCATAGAACAATGACTTGGGAGTTCA  192225

Query  22227   GAATCCTGGGCGTGAATAAGCCTCTGAGCCTTATTAAGCTGTGAAATCTAAAGCAAGACA  22286
               ||||||||||||||||||||||||||||||||||||||||||||||||||||||||||||
Sbjct  192224  GAATCCTGGGCGTGAATAAGCCTCTGAGCCTTATTAAGCTGTGAAATCTAAAGCAAGACA  192165

Query  22287   TTTCCCTGATAATTAAGCCTCATGAGCACTTATACTGTACACTTTACCTACATCAAATCA  22346
               |||| |||||||||||||||||||||||||||||||||||||||||||||||||||||||
Sbjct  192164  TTTCACTGATAATTAAGCCTCATGAGCACTTATACTGTACACTTTACCTACATCAAATCA  192105

Query  22347   TTCGGCCATCACCACAGACAAATGGCATGATTGATTTACTATCACCTTTTACTGGGCAGA  22406
               ||||||||||||||||||||||||||||||| |||||||||||||||||  |||||||||
Sbjct  192104  TTCGGCCATCACCACAGACAAATGGCATGATCGATTTACTATCACCTTTCGCTGGGCAGA  192045

Query  22407   AAAAGACAGAGATGAATGTGCCCAAGCTTATTAGCCCCTCAGCAAAAGAGCCAAGATGGG  22466
               |||||||||||||||||||||||||||||| |||||||||||||||||||||||||||||
Sbjct  192044  AAAAGACAGAGATGAATGTGCCCAAGCTTATGAGCCCCTCAGCAAAAGAGCCAAGATGGG  191985

Query  22467   AACCCAAGCATACAGCCCCAATGCTGAGGCTCTGAACTACTGACCTGCCCTCAGCACTCA  22526
               |||||||||||||||||||| ||| |||||||||||||||||| ||||||||||||||||
Sbjct  191984  AACCCAAGCATACAGCCCCAGTGCGGAGGCTCTGAACTACTGAGCTGCCCTCAGCACTCA  191925

Query  22527   GCCTTGGGATCATGAGTCACTGTGCAAGGGAGTCCCAACATCTGCATGTATGTCTGGAAT  22586
               ||||||||||||||||||||||||||||||||| ||||||||||||||||||||||||||
Sbjct  191924  GCCTTGGGATCATGAGTCACTGTGCAAGGGAGTTCCAACATCTGCATGTATGTCTGGAAT  191865

Query  22587   GATCTGAGCCTGTAGAGTTCCTACACACTGGCCACATTATAGGGTGGTGTCTGTGGTCAC  22646
               ||||||||||||||||||||||||||||||||||||||||||||||||||||||||||||
Sbjct  191864  GATCTGAGCCTGTAGAGTTCCTACACACTGGCCACATTATAGGGTGGTGTCTGTGGTCAC  191805

Query  22647   ACAGCTCAGGGCAGGTATTAGTACATGAATAGCTTAGCGGTGTCATAGTCTTTATGTGAA  22706
               |||||||||||||||||||||||||||||||||||||| |||||||||||||||||||||
Sbjct  191804  ACAGCTCAGGGCAGGTATTAGTACATGAATAGCTTAGCTGTGTCATAGTCTTTATGTGAA  191745

Query  22707   AGGCTCATAAAAGCCCAAATGCTTTTGAACTGGTTGCGGATTTTGAGTTGGAGGA--ACA  22764
               |||| ||||||||||||||||||||||||||||||||||||||||||||||||||  |||
Sbjct  191744  AGGCACATAAAAGCCCAAATGCTTTTGAACTGGTTGCGGATTTTGAGTTGGAGGACAACA  191685

Query  22765   CTTGGGACAACCGGTCACATTCCTTCAGTGCGCGGCCACTCCTCAGCACATATCCCAAAG  22824
               ||||||||||||||||||||||||||||||||||||||||||||||||||||||||||||
Sbjct  191684  CTTGGGACAACCGGTCACATTCCTTCAGTGCGCGGCCACTCCTCAGCACATATCCCAAAG  191625
```

```
Query  22825   CCGGTAAATAATGGGAAGACCCTGTACTGATGTTTGATGGTGTTATGAACTTCAAATGAC  22884
               ||||||||||||||||||||||||||||||||||||||||| ||||||||||||||||||
Sbjct  191624  CCGGTAAATAATGGGAAGACCCTGTACTGATGTTTGATGGTATTATGAACTTCAAATGAC  191565

Query  22885   CCCTATAGAAATGTTaaaaaaaaaaaaa--GTCTGTGAGCTACACAACCTGGGGACAAGA  22942
               ||||||||||||||||||||||||||||  ||||||||||||||||| ||||||||||||
Sbjct  191564  CCCTATAGAAATGTTAAAAAAAAAAAAAAAAGTCTGTGAGCTACACAAGCTGGGGACAAGA  191505

Query  22943   CAAG----AACACAAAATACCACTAAATAATGAGCCTCATGTGTCACCCTGCTTGCAACT  22998
               ||||    |||| |||||||||||||||||||||||||||||||||||||||||||||||
Sbjct  191504  CAAGTAAGAACATAAAATACCACTAAATAATGAGCCTCATGTGTCACCCTGCTTGCAACT  191445

Query  22999   CCCTTGGCAGCTGGTTTCAAGAGCCTCAGTGCTCACCAGGGCCCATCTCCCTGCTCTGCC  23058
               |||| |||||||||||| ||||||||||||||||||||||| ||||||||| ||||||||
Sbjct  191444  CCCTCGGCAGCTGGTTTGAAGAGCCTCAGTGCTCACCAGGGGCCATCTCCCCGCTCTGCC  191385

Query  23059   CTCTCTCGCTGTGGGTTGCCTGGGCTGAACATGGGCAGCCCCTCACACGATGACTGCAAA  23118
               |||||||||||||||||||||||||||||||| |||||||||||||| ||||||||||||
Sbjct  191384  CTCTCTCGCTGTGGGTTGCCTGGGCTGAACATAGGCAGCCCCTCACATGATGACTGCAAA  191325

Query  23119   CACAGGGCAAAGGAAGACTGAACGGAAAATGTATGGTTATAGTGGTTAttttaaaatata  23178
               ||||||||||||||||||||||||||||||||||||||||||||||||||||||||||||
Sbjct  191324  CACAGGGCAAAGGAAGACTGAACGGAAAATGTATGGTTATAGTGGTTATTTTAAAATATA  191265

Query  23179   agtatttcagtattttctaatctttaat---t-attattttaggggaggttttttttttt  23234
               ||||||||||||||||||||||||||||   | |||||||||||||||||||||||||||
Sbjct  191264  AGTATTTCAGTATTTTCTAATCTTTAATAATTGATTATTTTAGGGGAGGTTTTTTTTTTT  191205

Query  23235   ttt--GAGACAGAGTCTTGC--------CCAGGCTGGAGTGCAGTGGCATGATCTCAGCT  23284
               |||  |||||||||||||||        ||||||||||||||||||||||||||||||||
Sbjct  191204  TTTTTGAGACAGAGTCTTGCGCTGTTGCCCAGGCTGGAGTGCAGTGGCATGATCTCAGCT  191145

Query  23285   CACTGCAACCTCTGCCTCCTGTGTTCAAGCGATTCTCCTGCCTCAGCCTCCCAAGTAGCT  23344
               ||||||||||||| |||||||||||||||||||||||||||||||||||||| |||||||
Sbjct  191144  CACTGCAACCTCTACCTCCTGTGTTCAAGCGATTCTCCTGCCTCAGCCTCCCGAGTAGCT  191085

Query  23345   GGGACTACAGGCACGTGCCACCACGCCTGGCAAAGTTTTTGTGTTTTTAGTAGAGATGGG  23404
               |||| |||||||| ||||||||| |||||||||||||||||||||||||||||||| |||
Sbjct  191084  GGGAATACAGGCAAGTGCCACCATGCCTGGCAAAGTTTTTGTGTTTTTAGTAGAGACGGG  191025

Query  23405   GTTTCATTCTGTTAGCCAGGATGGTCTCAATCTTCTGTCCTCATGATCCACCCACCTCAG  23464
               |||||| |||||||||||||||||||||||||||||| ||||||||||||||| |||| |
Sbjct  191024  GTTTCACTCTGTTAGCCAGGATGGTCTCAATCTTCTGACCTCATGATCCACCCGCCTCGG  190965

Query  23465   CCTCCCAAAGTGCTGGGATTACAGGCGTGAGCCACCACACCTGGCCTGAttttttatttt  23524
               ||||||||||||||||||||||||||||||||||||||||||||||||| ||||||||||
Sbjct  190964  CCTCCCAAAGTGCTGGGATTACAGGCGTGAGCCACCACACCTGGCCTGAGTTTTTATTTT  190905

Query  23525   ttatttttttGAGATGAAGTCTCTGTCACCCAGGCTGGAGTGCAGTGGTGCAATCTCAGC  23584
               |  |||||||||||||||||||||||||||||||||||||||||||||||||||||||||
Sbjct  190904  TATTTTTTTTGAGATGAAGTCTCTGTCACCCAGGCTGGAGTGCAGTGGTGCAATCTCAGC  190845

Query  23585   TCACTGCAATCTCTGCCTCCAGGTTCAAGGGATTCTCCTGCCTCAGCCTCCTGAGTAGCT  23644
               ||||||||||||||||||||||||||||||||||||||||||||||||||||||||||||
Sbjct  190844  TCACTGCAATCTCTGCCTCCAGGTTCAAGGGATTCTCCTGCCTCAGCCTCCTGAGTAGCT  190785

Query  23645   GGGttttttttttttttttttttttttttttttttAGTTGAGATGGGGTTTACCATGTTG  23704
               |||||               |||||||||||||||| |||||||||||||||||||||||
Sbjct  190784  GGGTT--------------GTTTTTTTTTTTTTTTAATTGAGATGGGGTTTACCATGTTG  190739

Query  23705   GTCAGGCTGGTCTCGAACTCCTGACCTCAAATGATCCGCCCACCTCCACCTTCCAAACTG  23764
               ||||||||||||||||||||||||||||||||||||||||||||||||||||||||||||
Sbjct  190738  GTCAGGCTGGTCTCGAACTCCTGACCTCAAATGATCCGCCCACCTCCACCTTCCAAACTG  190679

Query  23765   CTGGGATTACAGGTGTGAGCCACTATGCCTGACT----ATTTTCATAACCAAGAAAAGAA  23820
               ||||||||||||||||||||||||||||||||||    ||||||||||||||||||||||
Sbjct  190678  CTGGGATTACAGGTGTGAGCCACTATGCCTGACTGATTATTTTCATAACCAAGAAAAGAA  190619

Query  23821   ATAAGTACAATTAATGCTGGTGCATGGTATTAAATCTAGTTTTTAAAAAATTCACACATA  23880
               |||| || ||||||||||||||||||||||||||||||||||||||||||||||||||||
Sbjct  190618  ATAAATATAATTAATGCTGGTGCATGGTATTAAATCTAGTTTTTAAAAAATTCACACATA  190559

Query  23881   AACAAGGCAGAACCCTATACCCTCCATGATAAATGCAGTAGCAGTGTATGTGGGTCTGTG  23940
                |||||||||||||||||||||||||||||||||||||||||||||||||||||||||||
Sbjct  190558  TACAAGGCAGAACCCTATACCCTCCATGATAAATGCAGTAGCAGTGTATGTGGGTCTGTG  190499

Query  23941   GAGGTTGAAAGGGACTTGGTAGATGTCAAGAAGGTAGTGGCAGTCTTGCTGGGCTTTTAA  24000
               ||||||||||||||||||||||||||||||||||||||||||||| ||||||||||||||
Sbjct  190498  GAGGTTGAAAGGGACTTGGTAGATGTCAAGAAGGTAGTGGCAGTCCTGCTGGGCTTTTAA  190439

Query  24001   AGGGTCTGAAGAAGTGACAGGATGCTGTGGTTGAATCCTAGCATGTATTTTAGCATTTGT  24060
               ||||||||||||||||||||||||||||||||||||||||||||||||||||||||||||
Sbjct  190438  AGGGTCTGAAGAAGTGACAGGATGCTGTGGTTGAATCCTAGCATGTATTTTAGCATTTGT  190379

Query  24061   TCATTTGGAGTTTGATTATTTCACGTTGCTTTCATTTGCCATTACCTGGAAAGCCAAGGG  24120
               ||||||||||||||||||||||||||||||||||||||||||||||||||||||||||||
Sbjct  190378  TCATTTGGAGTTTGATTATTTCACGTTGCTTTCATTTGCCATTACCTGGAAAGCCAAGGG  190319

Query  24121   CTCTACTCTCATTTCCTTGCTCCTGTTTCTTTGCCTTCCTTGGTCCGTGAAGAAGATGGT  24180
               ||||||||||||||||||||| || ||||||||||||||||||||| |||||||||||||
Sbjct  190318  CTCTACTCTCATTTCCTTGCTGCTCTTTCTTTGCCTTCCTTGGTCCATGAAGAAGATGGT  190259

Query  24181   CCAGGAGAAGCTCATTCCATGCTTGTTAACCAGGCACACCCCTAAGTTCCAGTCCCTGAG  24240
               ||||||||||||||||||||||||||||||||||||||||||||||||||||||||||||
Sbjct  190258  CCAGGAGAAGCTCATTCCATGCTTGTTAACCAGGCACACCCCTAAGTTCCAGTCCCTGAG  190199

Query  24241   TCATTCATGAGTAGCACTGCCAATGAACTGACAGCCATGCTGTGTCCCTCCACATCCCCT  24300
               ||||||||||||||||||||||||||||||||||||||||||||||||||||||||||||
Sbjct  190198  TCATTCATGAGTAGCACTGCCAATGAACTGACAGCCATGCTGTGTCCCTCCACATCCCCT  190139

Query  24301   AGGTGACTCGAAGAATTCTTCCAAAAAGCGTGTGAAAAGGGAGCCCTACTCTACTACCAA  24360
               |||||||||||||||  |||||||||||||||||||||||||||||||||||||||||||
Sbjct  190138  AGGTGACTCGAAGAAGCCTTCCAAAAAGCGTGTGAAAAGGGAGCCCTACTCTACTACCAA  190079

Query  24361   GGTAAAGCAGCCTGTCTTTGCCTAAGATGTAAATGTTGTTTTCTTGGATCCTTTATTTTT  24420
               ||||||| ||||||||||||||||||||||||||||||||||||||||||||||||||||
Sbjct  190078  GGTAAAGTAGCCTGTCTTTGCCTAAGATGTAAATGTTGTTTTCTTGGATCCTTTATTTTT  190019

Query  24421   CGGTTGATATCAGCTATGGGAAAATTATCCACTACATTATAGATGTTAGATAATATTTCC  24480
               | ||||||||||||||||||||||||||||||||||||||||||||||||||||||||||
Sbjct  190018  CAGTTGATATCAGCTATGGGAAAATTATCCACTACATTATAGATGTTAGATAATATTTCC  189959

Query  24481   TTGGGGATGGAGGAGGTGTATTTTACCAACTGACACCTGATTCCAGAGGACGTGCAAAAT  24540
               ||||||||||||||||||||||||||||||||||||||||||||||||||||||||||||
Sbjct  189958  TTGGGGATGGAGGAGGTGTATTTTACCAACTGACACCTGATTCCAGAGGACGTGCAAAAT  189899

Query  24541   TGGCAGTGTCAGATAATACACTGCGTGTTAAGGGATGTTTTCTTCAGGAACAAGCTTTCC  24600
               ||||||||||||||| ||||||||||||||||||||||||||||||||||||||||||||
Sbjct  189898  TGGCAGTGTCAGATAGTACACTGCGTGTTAAGGGATGTTTTCTTCAGGAACAAGCTTTCC  189839

Query  24601   ACTTTAGATAAGAATTCTTCAATTGCTACTCAAAAATTACCTAGACAGAAACATTCTTCA  24660
               |||||||||||||||||| |||||||||||||||||||||||||||||||||||||||||
Sbjct  189838  ACTTTAGATAAGAATTCTGCAATTGCTACTCAAAAATTACCTAGACAGAAACATTCTTCA  189779

Query  24661   AGAAAAGCTCCTGTGCTTTCCTAAGGGAACTCTACTCTAGAGTTGGGGCTTTTGACTTGA  24720
               ||||||||||||||||||||||||||||||||||||||||||||||||||||||||||||
Sbjct  189778  AGAAAAGCTCCTGTGCTTTCCTAAGGGAACTCTACTCTAGAGTTGGGGCTTTTGACTTGA  189719

Query  24721   ACCTTATTTCCAATCTTGGTTACCCAGAGTTTCCAAGTGAACAAAAGACCTGTGTGAGCC  24780
               ||||||||||||||||||||||||||||||||||||||||||||||||||||||||||||
Sbjct  189718  ACCTTATTTCCAATCTTGGTTACCCAGAGTTTCCAAGTGAACAAAAGACCTGTGTGAGCC  189659

Query  24781   ATCCATAGCATAGCCTGATTCTCAGAGTGTTTTCCTTCTCTAATTACAGGTGACTTCAGG  24840
               ||||||||||||||||||||||||||||||||||||||||||||||||||||||||||||
Sbjct  189658  ATCCATAGCATAGCCTGATTCTCAGAGTGTTTTCCTTCTCTAATTACAGGTGACTTCAGG  189599

Query  24841   GAGCACATTCAATGGTACGTATTCTGGAATCACTCACTGGTTGTTAGAAAAGGATTCTAC  24900
               ||||||||||||||||||||||||||||||||||||||||||||||||||||||||||| 
Sbjct  189598  GAGCACATTCAATGGTACGTATTCTGGAATCACTCACTGGTTGTTAGAAAAGGATTCTAT  189539

Query  24901   AGGAAATCTGGAGCTTAACTGCTGGCTTTTGTCTGGAGAGCCTCCATGATCCAAGATATC  24960
               |||||||||||||||||||||||||||||||||||||||||||||||||||||||| |||
Sbjct  189538  AGGAAATCTGGAGCTTAACTGCTGGCTTTTGTCTGGAGAGCCTCCATGATCCAAGACATC  189479

Query  24961   TTGTGGGAATGAGGATGTGGGGTATAGTAAAAGAAACTGGTTTTCCAGGTGACATACTCT  25020
               | ||||||||||||||||||| ||||||||| ||||||||||||||||||||||||||||
Sbjct  189478  TGGTGGGAATGAGGATGTGGGATATAGTAAAGGAAACTGGTTTTCCAGGTGACATACTCT  189419

Query  25021   TTTTATCTATTTATAGTTTCTGGGAACGTGTTCACATTAGGTtgtgtgtggttatgtgtg  25080
               |||||||||| ||||||||||||||| |||||||| ||||||||||||||| ||||||||
Sbjct  189418  TTTTATCTATGTATAGTTTCTGGGAATGTGTTCACGTTAGGTTGTGTGTGGGTATGTGTG  189359

Query  25081   tattagggcgggggtggggtgaggtggtctgtgtgCAAGTCTGCATGATTTGCTTGTGAA  25140
               |||||||||||||||||| ||||||||||||||||||  |||||||||||||||||||||
Sbjct  189358  TATTAGGGCGGGGGTGGGATGAGGTGGTCTGTGTGCACATCTGCATGATTTGCTTGTGAA  189299

Query  25141   TGTGTGTCTATGTGTGTTTTCCCTAGGaaaaaaaTGTTGTGTTTACCCAGCACAACTCTC  25200
               ||||||||||||||||||| ||| ||||||||||||||||||||||||||||||||||||
Sbjct  189298  TGTGTGTCTATGTGTGTTTCCCCCAGGAAAAAAATGTTGTGTTTACCCAGCACAACTCTC  189239

Query  25201   AGTGCCATTTTTCTTAATTTAACAAGTCAGACCACATACTTTACTTACATTAGTTCACAC  25260
               ||||||||||||||||||||||||| ||||||||||||||||||||||||||||||||||
Sbjct  189238  AGTGCCATTTTTCTTAATTTAACAAATCAGACCACATACTTTACTTACATTAGTTCACAC  189179

Query  25261   CTCATCATCATCATGCCCATATGTTGTGAGCTTGTTTATTGAGCCCACATGCCAGATGGA  25320
               ||||||||||||||||||||||||||||||||||||||||||| ||||||||||||||||
Sbjct  189178  CTCATCATCATCATGCCCATATGTTGTGAGCTTGTTTATTGAGTCCACATGCCAGATGGA  189119

Query  25321   GAAACTAAGCCACATAAATAAATGTGCCCTGGTTCACTTGCTGCATAGTGAAGAGTCAAA  25380
               ||||||||||||||||||||||||||||||||||||||||||||||||||||||||||||
Sbjct  189118  GAAACTAAGCCACATAAATAAATGTGCCCTGGTTCACTTGCTGCATAGTGAAGAGTCAAA  189059
```

```
Query  25381   ATGTTTACTCATATGGTGCTAATGTTGAAGGCCTGAACTACAACCTCTATTTATCAGCCA  25440
               ||||||||||||| ||||||||||||||||||||||||||||||||||||||||||||||
Sbjct  189058  ATGTTTACTCATACGGTGCTAATGTTGAAGGCCTGAACTACAACCTCTATTTATCAGCCA  188999

Query  25441   GTGAAGAGATCACTATTCACCATGCAAGGGAGTTCCAGCACCCTCTATGCCTGGAATTAC  25500
               ||||||||||||||||||||||||||||||||||||||||||||||||||||||||||||
Sbjct  188998  GTGAAGAGATCACTATTCACCATGCAAGGGAGTTCCAGCACCCTCTATGCCTGGAATTAC  188939

Query  25501   CCACGCCTGCAGAGATCCCAAACGCCACCCCTCACATAAGAGAGCCTCATGATCTCATAA  25560
               ||||||||||||||||||||||||||| ||||||||||||||||||||||||||||||||
Sbjct  188938  CCACGCCTGCAGAGATCCCAAACGCCATCCCTCACATAAGAGAGCCTCATGATCTCATAA  188879

Query  25561   TCCAGGTAGCTATGTAGACACCTTCCTGCAGGTGTCACATAGTCCTTTGTGTGAAACCAA  25620
               |||||||||||||||||||| |||||||||||||||||||||||||| ||| ||||||||
Sbjct  188878  TCCAGGTAGCTATGTAGACATCTTCCTGCAGGTGTCACATAGTCCTTAGTGGGAAACCAA  188819

Query  25621   CATAGAAAGCCCATGTTTCTGATCAAATCACAGGTTCTGAAACACTAAGGGAGGCACTAA  25680
               |||||||||||||||||||||||||||||||||||||||||||||||||||| |||||||
Sbjct  188818  CATAGAAAGCCCATGTTTCTGATCAAATCACAGGTTCTGAAACACTAAGGGAAGCACTAA  188759

Query  25681   GTAGGACAACGTGGTGCCTGCGTGTCATAGCTGGGTCTCCTCAAGACATGGATCAAGTCC  25740
               ||||||||| |||||||||| |||||||||||||||||||||||||||||||||||||||
Sbjct  188758  GTAGGACAATGTGGTGCCTGTGTGTCATAGCTGGGTCTCCTCAAGACATGGATCAAGTCC  188699

Query  25741   AGTAAGAATTGGGGAGACGCTTTAGAGTCTTGATGGAGTTATCACCACAAGCCCTCTGAG  25800
               | |||||||||||||||| |||||||||||||||||||||||||||||||||||||||||
Sbjct  188698  AATAAGAATTGGGGAGATGCTTTAGAGTCTTGATGGAGTTATCACCACAAGCCCTCTGAG  188639

Query  25801   CTACACACATTAGGGATCATGACCATTAAGTACTCAAATTACCATTTGGTTGTTATCCGG  25860
               |||||||| ||||||||||||||||||||||||||||||||||| |||||||||||||||
Sbjct  188638  CTACACACTTTAGGGATCATGACCATTAAGTACTCAAATTACCACTTGGTTGTTATCCGG  188579

Query  25861   GTATCTGTCGTCCTTGTGGCAACCCTCTTGTGAAGCTGGTGTGGACAGCCTCAGTGCTGG  25920
               ||||| ||||||||||||||||| ||||||||||||||||||||||||||||||||||||
Sbjct  188578  GTATCCGTCGTCCTTGTGGCAACTCTCTTGTGAAGCTGGTGTGGACAGCCTCAGTGCTGG  188519

Query  25921   AGCTGTGCCTCCCTTCTGAGTGGACCCTTTCTGTGTTAGCAGGTGGGTACAAGCGTGGGG  25980
               |||||||||| |||||||| |||||||||||||||||||||||||||||||||| |||||
Sbjct  188518  AGCTGTGCCTGCCTTCTGAATGGACCCTTTCTGTGTTAGCAGGTGGGTACAAGCATGGGG  188459

Query  25981   GTCAGCACACTCAGTGGATTTACACACACAGCATTAAAGAGTAAGGCTGCGCTT---TGT  26037
               ||||||||||||||||||||||||||||||||||| ||||||||||||| ||||   | |
Sbjct  188458  GTCAGCACACTCAGTGGATTTACACACACAGCATTGAAGAGTAAGGCTGGGCTTCATTAT  188399

Query  26038   TTATACATTTTCAATAAATGATGATCTTCATAACATAAAATCAATGATGTAGTACACTAG  26097
               ||||||||||||||||||||||||||||||||||||||||||||||| ||||||||||||
Sbjct  188398  TTATACATTTTCAATAAATGATGATCTTCATAACATAAAATCAATGACGTAGTACACTAG  188339

Query  26098   AATACTGTCCCTAGTATTGAATCTTGTCTCTCAACAAAGGGTTGCTTAAAGTCACATGAC  26157
               ||||||||||||||||||||||||||||||||||||||||||||||||||||||||||||
Sbjct  188338  AATACTGTCCCTAGTATTGAATCTTGTCTCTCAACAAAGGGTTGCTTAAAGTCACATGAC  188279

Query  26158   AGATTCCATTCAACTGATGACACATGCTGTAGCAGCAGTTAAAGCAGTCATTTGAAAAGG  26217
               ||||||||||||||||||||||||||||||||||||||||||||||||||||||||||||
Sbjct  188278  AGATTCCATTCAACTGATGACACATGCTGTAGCAGCAGTTAAAGCAGTCATTTGAAAAGG  188219

Query  26218   CTTTTACTATAAACTTATGTGTGAGCCTGAAGTGGGGGATAAAAGAGGTGATTAGCTACC  26277
               ||||||||||||||||||||||||||||||||||||||||||||||||||||||||| ||
Sbjct  188218  CTTTTACTATAAACTTATGTGTGAGCCTGAAGTGGGGGATAAAAGAGGTGATTAGCTCCC  188159

Query  26278   CTGTGCCATGTTTCTATTATGTGTGTGGTGGAGGAAAATTACACAGGAAGGTGATGGAGA  26337
               |||||||||||||||||||||||  |||||||||||||||||||||||||||||||||||
Sbjct  188158  CTGTGCCATGTTTCTATTATGTGCATGGTGGAGGAAAATTACACAGGAAGGTGATGGAGA  188099

Query  26338   GAACAGAGCAAAGGATTGGACAGGTCCATTGAACCCATAAGACTATGTTGAGGTTAGTGA  26397
               ||||||||||||||||||||||||||||||||||||||||||||||| ||||||||||||
Sbjct  188098  GAACAGAGCAAAGGATTGGACAGGTCCATTGAACCCATAAGACTATGGTGAGGTTAGTGA  188039

Query  26398   ATGAGACTGGTCATTTTAGGTCAAATTTTACCCAGAGCTGGTGCAACCACTGCCCATTCT  26457
               |||||| |||||||||||||||||||||||||||||||||||||| ||||||||||||||
Sbjct  188038  ATGAGATTGGTCATTTTAGGTCAAATTTTACCCAGAGCTGGTGCAGCCACTGCCCATTCT  187979

Query  26458   TAGCCAGACCTTATTGCAGGTAGCTCTGATCAATAGTCAAGGAGGCAGTGGGGGTTGCAG  26517
               |||||||||||||||||||| ||||| |||||||||||||||||||||||||||||||||
Sbjct  187978  TAGCCAGACCTTATTGCAGGCAGCTCCGATCAATAGTCAAGGAGGCAGTGGGGGTTGCAG  187919

Query  26518   ACTTAATTCATTAAATCACCAAAGCACCAGCCCACATGGCCACTTTTCCAGTTAATTCAC  26577
               |||||||||||||||||||||||||||||||||||| |||||||||||||||||||||||
Sbjct  187918  ACTTAATTCATTAAATCACCAAAGCACCAGCCCACACGGCCACTTTTCCAGTTAATTCAC  187859

Query  26578   AGTAGCTTGCATATTCAGGTTTGATCATTGGAAGCGAAGTTACTCTTTGCAGACCCATCT  26637
               ||||||||||||||||||||||||||| |||||| |||||||||||||||||||||||||
Sbjct  187858  AGTAGCTTGCATATTCAGGTTTGATCAGTGGAAGGGAAGTTACTCTTTGCAGACCCATCT  187799

Query  26638   TTTGACAATCATTTTGCAGTGTCAGAAGGTCTGAGCAGCCTCGGGAGGCAAGCAGTCCCT  26697
               ||||||||||||||||||||||||||||||||||||||||||||||||||||||||||||
Sbjct  187798  TTTGACAATCATTTTGCAGTGTCAGAAGGTCTGAGCAGCCTCGGGAGGCAAGCAGTCCCT  187739

Query  26698   GGTCCCTCAGTGTAGTCACTGGAGGAGACAGTCACTGAGAGGCAGCTGGCAGGGTGAAGG  26757
               ||||||||||||||||||||||||||||||||||||||||||||||||||||||||||||
Sbjct  187738  GGTCCCTCAGTGTAGTCACTGGAGGAGACAGTCACTGAGAGGCAGCTGGCAGGGTGAAGG  187679

Query  26758   GAAAGGGGAGGCAGGCCACAGAGATGACAGCCTTTAAGCTGTCATACTGGGGAAGTCAAG  26817
               ||||||||||||||||||||||||||||||||||||||||||||||||||| | ||||||
Sbjct  187678  GAAAGGGGAGGCAGGCCACAGAGATGACAGCCTTTAAGCTGTCATACTGGG-AGGTCAAG  187620

Query  26818   GATCTGAAAGAGGAAGGAGAATTCTTTATCATTAAGGACCTGTCCTTATCTCAGGCATTT  26877
               ||||||||||||||||||||||||||||||||||||||||||||||||||||||||||||
Sbjct  187619  GATCTGAAAGAGGAAGGAGAATTCTTTATCATTAAGGACCTGTCCTTATCTCAGGCATTT  187560

Query  26878   CCTCCAGAGCATCACCTTTGTCCACCCACACACCTTGGGCTAGGAGGACTGGGGAAAGAC  26937
               ||||||||||||||||||||||||||||||||||||||||||||||||||||| ||||||
Sbjct  187559  CCTCCAGAGCATCACCTTTGTCCACCCACACACCTTGGGCTAGGAGGACTGGAGAAAGAC  187500

Query  26938   AGTGAAGGGCCTCTTGGGTCTCTGGCACAGGGCGTGATGAAGAGGTGGCAGTTTTTCAGG  26997
               ||||| ||| ||||||||||||||||||||||||||||||||||||||||||||||||||
Sbjct  187499  AGTGAGGGGTCTCTTGGGTCTCTGGCACAGGGCGTGATGAAGAGGTGGCAGTTTTTCAGG  187440

Query  26998   AAACTCTCTCTCTAGGAACCAAATACATTTCCCATCTCAGGTCCTTCACTCAGCGGGGT  27057
               || ||||||||||||||||||||||||||||||||||||| |||||||||||||||||||
Sbjct  187439  AATCTCTCTCTCTAGGAACCAAATACATTTCCCATCTCAGCTCCTTCACTCAGCGGGGT  187380

Query  27058   TGAGGTTCTGCTCGTCACTTATCATCTCTGAATGTCAGCACCCTCAAGTGTAAAATCTCA  27117
               ||||||||||||||||||||||||||||||||||||||||||||||||||||||||||||
Sbjct  187379  TGAGGTTCTGCTCGTCACTTATCATCTCTGAATGTCAGCACCCTCAAGTGTAAAATCTCA  187320

Query  27118   GCCACAGCCCCTCCTCTGCACCCCCTGCAGGGCTGATGTTCTCCATAAACCATAAGGCAT  27177
               ||||||||||||||||||||||||||||||||||||||||||||||||||||||||||||
Sbjct  187319  GCCACAGCCCCTCCTCTGCACCCCCTGCAGGGCTGATGTTCTCCATAAACCATAAGGCAT  187260

Query  27178   CATGCCCACGGAAAAGCTGAACAGGAAAGCATGCTCCACTGCCCCGGAGCCATCCAAGTT  27237
               ||||||||||||||||| ||||||||||||||||||||||||||| ||||||||||||||
Sbjct  187259  CATGCCCACGGAAAAGCCGAACAGGAAAGCATGCTCCACTGCCCCAGAGCCATCCAAGTT  187200

Query  27238   CCCCCTCCATATTCCACCACTGCTAAGTGTCCAGCTTATTCCTCCTGGCATGTAGTAAAC  27297
               ||||||||||||||| ||||||||||||||||||||||||||||||||||||||||||||
Sbjct  187199  CCCCCTCCATATTCCGCCACTGCTAAGTGTCCAGCTTATTCCTCCTGGCATGTAGTAAAC  187140

Query  27298   ACTTAGAGAACATTACTGAAGTACCAGTCCTCTCTAAGGTTTTCCTGTATTTAGTGATTT  27357
               ||||||||||||||||||||||||||||||||||||||||||||||||||||||||||||
Sbjct  187139  ACTTAGAGAACATTACTGAAGTACCAGTCCTCTCTAAGGTTTTCCTGTATTTAGTGATTT  187080

Query  27358   TTTAGCCCCGTACTGTGATACTAAGAAGTAGGGCCTAAATAGGGCCTAAAAAGTATTGCT  27417
               |||||||| |||||||||||||||||||||||||||||||||||||||||||||||||||
Sbjct  187079  TTTAGCCCTGTACTGTGATACTAAGAAGTAGGGCCTAAATAGGGCCTAAAAAGTATTGCT  187020

Query  27418   AAAATTACATTATGACAGTGCAGAGAACTGAGGGCAGAGGGAGGACATGAGATTTCTAGG  27477
               ||||||||||||||||||||||||||||||||||||||||||||||||||| || | |||
Sbjct  187019  AAAATTACATTATGACAGTGCAGAGAACTGAGGGCAGAGGGAGGACATGAGCTTGCCAGG  186960

Query  27478   TCCACATGGCTTAGTGGAATTTGAATCCGGGCCCCCACTCTGCACCAGCCCTGCACTCAC  27537
               ||||||||||||||||||||||||||||||||||||||||||||||||||||||||||||
Sbjct  186959  TCCACATGGCTTAGTGGAATTTGAATCCGGGCCCCCACTCTGCACCAGCCCTGCACTCAC  186900

Query  27538   AGTCATCCTGCTGTGTTCTCCTCTCCAGGAAGGCACTGCCCACACAGTCTGTCTGATAGA  27597
               |||||||||||||| |||||||||||||||||||||||||||| ||||||||||||||||
Sbjct  186899  AGTCATCCTGCTGTATTCTCCTCTCCAGGAAGGCACTGCCCACGCAGTCTGTCTGATAGA  186840

Query  27598   GGTGTTGAGTGCTCACTGAACTCCGTGATCTTCCTGAAACCCAACTTTGATTCAGTGGGC  27657
               ||||||||||||||||||||||||||||||||||||||||||||||||||||||||||||
Sbjct  186839  GGTGTTGAGTGCTCACTGAACTCCGTGATCTTCCTGAAACCCAACTTTGATTCAGTGGGC  186780

Query  27658   TCTGCTTGGAAGCCTGTAAAGAAAAACATCATAAGTTTAAACTTAGAACAGATTATCACT  27717
               |||||||||||||||||||||||||  |||||||||||||||||||||||||||||||||
Sbjct  186779  TCTGCTTGGAAGCCTGTAAAGAAAAGGATCATAAGTTTAAACTTAGAACAGATTATCACT  186720

Query  27718   GTTTTCCCTCTGGTCTTCAGTCAGCAAGATGTCAACAGCCCTATCTATTGTAAATGCGTT  27777
                ||||||||||||||||| |||||||||||||||||||||||||||||||||||||| ||
Sbjct  186719  ATTTTCCCTCTGGTCTTCTGTCAGCAAGATGTCAACAGCCCTATCTATTGTAAATGCATT  186660

Query  27778   AACCAGCATCTTCTCTGATAGAGAATACAAGAAGATATGCTGTGCACACCAACCAGTGTC  27837
               ||||||||||||||||||||||||||||||||||||||||||||||||||||||||||||
Sbjct  186659  AACCAGCATCTTCTCTGATAGAGAATACAAGAAGATATGCTGTGCACACCAACCAGTGTC  186600

Query  27838   GGAGACCTCATGGCTCCCCGGTAAAGAAGAAGATGTACCCACAAGAAGGTACTGTGGAAG  27897
               ||||||||||||||||||||||||||||||||| ||||||||||||||||||||||||||
Sbjct  186599  GGAGACCTCATGGCTCCCCGGTAAAGAAGAAGAGGTACCCACAAGAAGGTACTGTGGAAG  186540

Query  27898   TTCATTAATTAAGTTGCTTCAAGAATTGCAATTGCGGGGAGTATTCAGTGTCCCATATGT  27957
               |||||||||||||||| |||||||||||||||||||||||||||||||||||||||||||
```

```
Sbjct  186539  TTCATTAATTAAGTTGATTCAAGAATTGCAATTGCGGGGAGTATTCAGTGTCCCATATGT  186480

Query  27958   AAGAGGAAACTATGAAGAGACTAAGCCATATTTTTAATGTGTCAGGATTCTAATTTGCC  28017
               ||||||||||||||||||||||||||||||||||||||||||||||||||||||||||||
Sbjct  186479  AAGAGGAAACTATGAAGAGACTAAGCCATATTTTTAATGTGTCAGGATTCTAATTTGCC  186420

Query  28018   TGGTCAGTAAATATTGCTACAACCACAAAAGTAAATATCTACTTAAAAGTCAATTGTGGT  28077
               |||||||||||||||||||| |||||||||||||||||||||||||||||||||| ||||
Sbjct  186419  TGGTCAGTAAATATTGCTACCACCACAAAAGTAAATATCTACTTAAAAGTCAATTTTGGT  186360

Query  28078   TCATGTTTAATGATAGACAATGTTTCAAGCTAATGTCTAGAACTTACCTGGTTGTTAAAC  28137
               ||||||||||||||||||||||||||||||||||||||||||||||||||||||||||||
Sbjct  186359  TCATGTTTAATGATAGACAATGTTTCAAGCTAATGTCTAGAACTTACCTGGTTGTTAAAC  186300

Query  28138   ATAAGCATAGATCTCCCTGAAAGAGTGGTGCTATATTATTATTTTTCAATTAATATATTT  28197
               ||||||||||||||||||||||||||||||||||||||||||||||||||||||||||||
Sbjct  186299  ATAAGCATAGATCTCCCTGAAAGAGTGGTGCTATATTATTATTTTTCAATTAATATATTT  186240

Query  28198   CTTTAGAGAGTTTTAAATTGACATAAAAACTGAGCATATGGCCAGGCGTGGTGGCTCACG  28257
               ||||||||||||||||||||||||||||||||||||||||||  |||||||||||||||
Sbjct  186239  CTTTAGAGAGTTTTAAATTGACATAAAAACTGAGCATATGGCTGGGCGTGGTGGCTCACA  186180

Query  28258   CTTATAAACCCAGCACTTTAGGAGGCCAAGGCAGGCGGATCATCTGAGGTCAGGAGTTGG  28317
               ||||||| ||||||||||||||||||||||||||||||||||||||||||||||||||||
Sbjct  186179  CTTATAATCCCAGCACTTTAGGAGGCCAAGGCAGGCGGATCATCTGAGGTCAGGAGTTGG  186120

Query  28318   AGACCAGCCTGGCCAACATGGTGAAACCCCATCTCTACTAAAAATACaaaaaaaTTAGCC  28377
               ||||||||||||||||||||||||||||||||||||||||||||||||||||||||||||
Sbjct  186119  AGACCAGCCTGGCCAACATGGTGAAACCCCATCTCTACTAAAAATACAAAAAAATTAGCC  186060

Query  28378   CGGTATGGTGGCAGGCGCCTGTAATCCCAGCTACTCAGGAGGCTGAGGCAGGAGAATCGC  28437
                ||| |||||||||| ||||||||||||||||||||||||||||||||||||||||||||
Sbjct  186059  GGGTGTGGTGGCAGGTGCCTGTAATCCCAGCTACTCAGGAGGCTGAGGCAGGAGAATCGC  186000

Query  28438   TTGAACCCGGGAGGCAGAGGTTGCAGTGAGCCAAGATCATGCCATTGCACTCCAGCCTGG  28497
               |||||||| |||||||||||||||||||||||||||||||||||||||||||||||||||
Sbjct  185999  TTGAACCCAGGAGGCAGAGGTTGCAGTGAGCCAAGATCATGCCATTGCACTCCAGCCTGG  185940

Query  28498   GTGACAAGAGTGAAACTCCATCTCaaaataaataaataaataaataaataaataaataaa  28557
               ||||||||||||||||||||||||||||||||||||||||||||||||||||
Sbjct  185939  GTGACAAGAGTGAAACTCCATCTCAAAATAAATAAATAAATAAATAAATAAA--------  185888

Query  28558   taaaaatTGAGTATATAATACACGAAGTTCCCATATTATTCTGTCTCCTCATCCTCACTT  28617
                   ||||||||||||||||||| ||||||||||||||||||||||||||| ||||||||
Sbjct  185887  ----AATTGAGTATATAATACACAAAGTTCCCATATTATTCTGTCTCCTCACCCTCACTT  185832

Query  28618   CCTAATTTCACCTATTAGTAACATCTTACATTACTGTGGTACATTTGCTAGAATAATGAG  28677
               ||||||||||||||||||||||||||||||||||||||||||||||||||||||||||||
Sbjct  185831  CCTAATTTCACCTATTAGTAACATCTTACATTACTGTGGTACATTTGCTAGAATAATGAG  185772

Query  28678   AAAATATTGATACATTATTATCTAAAGTCTACATTTGCATAATGTTCATTCTTTCTGTTA  28737
               || ||||||| |||||||||||| ||||||||||||||||||||||||||||||||||||
Sbjct  185771  AACATATTGACACATTATTATCTGAAGTCTACATTTGCATAATGTTCATTCTTTCTGTTA  185712

Query  28738   TACATATATATGAATTTTGAAATATTTAAAACATTATGTTCACCCTTATGGTCTCATAAA  28797
               ||||||||||||||||||||||||||||||||||||||||||||||||||||||||||||
Sbjct  185711  TACATATATATGAATTTTGAAATATTTAAAACATTATGTTCACCCTTATGGTCTCATAAA  185652

Query  28798   GAAAATGTTCACTTCCCTAAAATCCTCTCTTCTCATTAATCTCTGTCCTCTTTCTCCAG  28857
               |||||||||||||||||||||||||| |||||||||||||||||||||||||||||||||
Sbjct  185651  GAAAATGTTCACTTCCCTAAAATCCTGTCTTCTCATTAATCTCTGTCCTCTTTCTCCAG  185592

Query  28858   AAACCATGGCAACTATTAACATTTTTACTATCGCTTCAACTTTGCCTTTTCCAGAATGTC  28917
               ||||| ||||||||||||| |||||||||||||||||| |||||||||||||||||||||
Sbjct  185591  AAACCTTGGCAACTATTAATATTTTTACTATCGCTTCAGCTTTGCCTTTTCCAGAATGTC  185532

Query  28918   GTATAGTTGGAATCATATATTATGTAGTTTTTCAGATGAATTTATTGCACTAAATTGAT  28977
                |||||||||||||||||||||||||||||||||||||||||||||| ||||||||||||
Sbjct  185531  ATATAGTTGGAATCATATATTATGTAGTTTTTCAGATGAATTTATTGTACTAAATTGAT  185472

Query  28978   GTACGCTTTAGCTGCTTTCATGTCTTTTTTATGCCTTAATGGCAAAAAATGGCACATTAA  29037
               ||||||||||||||||||||||||||||||||||||||||||||||||||||||||||||
Sbjct  185471  GTACGCTTTAGCTGCTTTCATGTCTTTTTTATGCCTTAATGGCAAAAAATGGCACATTAA  185412

Query  29038   ATCACCAAATAATATTGCATTAAATGAATTTTTGTCTTTTTATTCGCCTGTTGAAGAATT  29097
               ||||||||||||||||||||||||||||||||||||||||||||| ||||||||||||||
Sbjct  185411  ATCACCAAATAATATTGCATTAAATGAATTTTTGTCTTTTTATTCACCTGTTGAAGAATT  185352

Query  29098   CGGTAGATTTCATGAGAGAAACCATCTGGGCCTGGTGCTTTCTTTTTCGGAATGCTCTTA  29157
               |||||||||||||||||||||||||||||||||||||||||||||||| |||||||||||
Sbjct  185351  CGGTAGATTTCATGAGAGAAACCATCTGGGCCTGGTGCTTTCTTTTTCAGAATGCTCTTA  185292

Query  29158   ATGTGAATTCAACTTATTTAATAGACATAAGTTATTCACATTAGGATTCTAGCGTGACC  29217
               ||||||||| |||||||||||||||||||||||||||| |||||||| ||||| |||||
Sbjct  185291  ATGTGAATTGAACTTATTTAATAGACATAAGTTATTCAAATTAGGATCCTAGCATGACC  185232

Query  29218   TTGGGAAGATTGCCTTTCAAGGAATTGATAAATTTCACTGAGGTTATCAAACTGCGGTCA  29277
               |||||||||||||||||||||||||||||| |||||||||||||||||||||||||||||
Sbjct  185231  TTGGGAAGATTGCCTTTCAAGGAATTGATACATTTCACTGAGGTTATCAAACTGCGGTCA  185172

Query  29278   TAGAACTGTTCATAATATTCCTTTTAATGCCTAACAGTTCAGTAGAGATGGCTCCTCTTT  29337
               ||||| ||||||| ||||||||||||||||||||||||||||||||||||||||||||||
Sbjct  185171  TAGAAATGTTCATGATATTCCTTTTAATGCCTAACAGTTCAGTAGAGATGGCTCCTCTTT  185112

Query  29338   TATTTCTGAAATTGGTCATTTGTGTTATCTTCTTTTTCTTGGTTAGCCTGCATATCAATT  29397
               ||||||||||||||||||||||||||||||||||||||||||||||||||||||||||||
Sbjct  185111  TATTTCTGAAATTGGTCATTTGTGTTATCTTCTTTTTCTTGGTTAGCCTGCATATCAATT  185052

Query  29398   CATTCATTGTAATGAGCATATCAAAGAACCAGCttttggttttattgattttctgatgat  29457
               ||||||||||||||||||||||||||||||||||||||||||||||||||||||||||||
Sbjct  185051  CATTCATTGTAATGAGCATATCAAAGAACCAGCTTTTGGTTTTATTGATTTTCTGATGAT  184992

Query  29458   ttcagtgttttaattttattgatttctgtgatgttgtttattaCTTTTACTTGCTTTCCA  29517
               ||||||||||||||||||||||||||||||||||||||||||||||||||||||||||||
Sbjct  184991  TTCAGTGTTTTAATTTTATTGATTTCTGTGATGTTGTTTATTACTTTTACTTGCTTTCCA  184932

Query  29518   TTGCATTCCTCTATTTTCTACAGTTCCCTAATTGAAACATGATATTACTGATTTTAGATC  29577
               |||||||||||||||||||| |||||||||||||||||||||||||||||||||||| ||
Sbjct  184931  TTGCATTCCTCTATTTTCTATAGTTCCCTAATTGAAACATGATATTACTGATTTTAGGTC  184872

Query  29578   TTGTGATTTTTAGTATATTACATCCAATGCTATAGATTTCCCTCTAAGGACTGCTTTTGC  29637
               ||||||||||||||||||| ||||||||||||||||||||||||||||||||||||||||
Sbjct  184871  TTGTGATTTTTAGTATATTGCATCCAATGCTATAGATTTCCCTCTAAGGACTGCTTTTGC  184812

Query  29638   TACATCCAGAAATCTTGCCAAGTCACATTTTCTTTTAATGTAGTTAAAAGTATTTTTAAT  29697
               ||||||||||||||||||||||||||||||||||||||||||||||||||||||||||||
Sbjct  184811  TACATCCAGAAATCTTGCCAAGTCACATTTTCTTTTAATGTAGTTAAAAGTATTTTTAAT  184752

Query  29698   TTTCTATTGAGACTTCTTCTTTAACCCATGAGTTATTTAAAACTGCATTGCTAATTTGCA  29757
               |||||||||||||||||||||||||||||||||||||||||| |||||||||||||||||
Sbjct  184751  TTTCTATTGAGACTTCTTCTTTAACCCATGAGTTATTTAAAAGTGCATTGCTAATTTGCA  184692

Query  29758   AATATTTGGGGATTTTGTGGCTCTTTTACAGTTGTTGATTTTTTGTTGTCAGGTATGTGT  29817
               ||||||||||||||||||||||||||||||||||||||||||||||||||||||||||||
Sbjct  184691  AATATTTGGGGATTTTGTGGCTCTTTTACAGTTGTTGATTTTTTGTTGTCAGGTATGTGT  184632

Query  29818   TGCAAAAGCAGTCATCTACCTCATCTTGCCACCACCCAAGATGGCCCAGGATGTGGGCTC  29877
               ||||||||||||| ||||||||||||||||||||||||||||||||||||||||||||||
Sbjct  184631  TGCAAAAGCAGTCGTCTACCTCATCTTGCCACCACCCAAGATGGCCCAGGATGTGGGCTC  184572

Query  29878   TCCCTGAGTGAATCTTTGGCAATCTGCCAACCTGATGTGGTCGGCCTCCTTCTTTAGTCT  29937
               ||||||||||||||||||||||||||||||||||||| | |||||||||||   ||||||
Sbjct  184571  TCCCTGAGTGAATCTTTGGCAATCTGCCAACCTGATGCGTTCGGCCTCCTT---TAGTCT  184515

Query  29938   GAGCTTGTCTTCTGCTTAGAAAGGGCCATTCTCAGTTCTGGCAGGGAGTTTTCCCAACAT  29997
               ||||||| |||||||||||||||||||||||||||||||||||||||||| |||||||||
Sbjct  184514  GAGCTTGCCTTCTGCTTAGAAAGGGCCATTCTCAGTTCTGGCAGGGAGTTTCCCCAACAT  184455

Query  29998   TGAGAAGGTGGCATTCTTACTCCCCAGTGCAGCCTGCACCTCTGACCGGTGGTCAGCAGA  30057
               ||||||||||||||||||||||||||| ||||||||||||||||||||||||||||||||
Sbjct  184454  TGAGAAGGTGGCATTCTTACTCCCCACTGCAGCCTGCACCTCTGACCGGTGGTCAGCAGA  184395

Query  30058   CAGGACAGAGGTCCTCATTAGACAGAGTTCAGCGGGGTCTCTGACCAAAGGGCATCTTCA  30117
               |||||||||||||||||||||||||||||||||||||||||||||||||| |||||||||
Sbjct  184394  CAGGACAGAGGTCCTCATTAGACAGAGTTCAGCGGGGTCTCTGACCAAAGTGCATCTTCA  184335

Query  30118   GAGTCTGCACCTACCCACTGTGACCATGGGCAGGCTCTGAGTCCTAAAGCAGGAGGAACC  30177
               |||||||||||||||||||||||||| ||||||||||||||||||||||||||||||||
Sbjct  184334  GAGTCTGCACCTACCCACTGTGACCACGGGCAGGCTCTGAGTCCTAAAGCAGGAGGAACT  184275

Query  30178   GTGCGACCATCCTGATTGGAAATTTGTGAGGATCACCATGTTACTCAAGTAAGGTCTTTG  30237
               ||||||||||||||||||||||||||||||||||||||||||||||||||||||||||||
Sbjct  184274  GTGCGACCATCCTGATTGGAAATTTGTGAGGATCACCATGTTACTCAAGTAAGGTCTTTG  184215

Query  30238   GAAAGTGTCGTATTACTACTGTTTGTGAACTTCTTGTTGGTGGCCTGGCTGAGCCACACA  30297
               ||||||||||||||||||||||||||||||| ||||||||||||||||||||||||||||
Sbjct  184214  GAAAGTGTCGTATTACTACTGTTTGTGAACTGCTTGTTGGTGGCCTGGCTGAGCCACACA  184155

Query  30298   CTTTATGAAAACCAGGACCCCTCAGCTGGTGTGGGTGTCTATGCAGCCTGAGACCCTCAT  30357
               ||||||||||||||||||||||||||||||||||||||||||||||||||||||||||||
Sbjct  184154  CTTTATGAAAACCAGGACCCCTCAGCTGGTGTGGGTGTCTATGCAGCCTGAGACCCTCAT  184095

Query  30358   GTGAACAGCCTCGTGGTAGCTGTCTTTGCCCCTTGCCACCATCAGTGCCTCCTTGTTCCT  30417
               |||||||||||||||| |||||||||||||||||||||||||||||||||||||||||||
Sbjct  184094  GTGAACAGCCTCGTGGCAGCTGTCTTTGCCCCTTGCCACCATCAGTGCCTCCTTGTTCCT  184035

Query  30418   GGGCACTGCTTTCTCTGATGGTGCTCCATTGTTTTCCTGCACCTCAGTGTCTACAGCTGG  30477
               ||||||||||||||||||||||||||||||| ||||||||||||||||||||||||||||
Sbjct  184034  GGGCACTGCTTTCTCTGATGGTGCTCCATTTTTTTCCTGCACCTCAGTGTCTACAGCTGG  183975

Query  30478   ATGTCTCTTCTGCAATCTAGGTGAGGGGGCATCAATAACAGTTCTGCTGTGGCACTGCCC  30537
```

```
               |||||||||| |||||||||| ||||||||||||||  ||||||||||||||||||||||
Sbjct  183974  ATGTCTCTTCCGCAATCTAGGCGAGGGGGCATCAATGGCAGTTCTGCTGTGGCACTGCCC  183915

Query  30538   TCCTTCTTAGCTTGTCTTGCTCCGTCTTAGGCTCCCTCAAAGATCCCACCCTTCAGGTTC  30597
               |||||||||||||||||||||| |||||||||||||| ||||||||||||||||||||||
Sbjct  183914  TCCTTCTTAGCTTGTCTTGCTCTGTCTTAGGCTCCCTGAAAGATCCCACCCTTCAGGTTC  183855

Query  30598   TTCCACAAGTTTCTTATTGAAATCCGAGCAGAAAACTATGACCAATATGACCAATCGTAT  30657
               |||||||||||||||||||||||||||||||||||||||||||||||||||||||| |||
Sbjct  183854  TTCCACAAGTTTCTTATTGAAATCCGAGCAGAAAACTATGACCAATATGACCAATCCTAT  183795

Query  30658   ACCCACTGAAGACATGAATGTAGAATTAAAACAATTCTCCATGGACTCTACCATATAGAT  30717
               |||||||||||||||||||| |||||||||||||||||||||||||||||||||||||||
Sbjct  183794  ACCCACTGAAGACATGAATGAAGAATTAAAACAATTCTCCATGGACTCTACCATATAGAT  183735

Query  30718   CCCTAGAAGTAATTTCTaaaaaaaaaaaaaaa---TCAAGGAAGATGTAATAGTTTTCCA  30774
               ||||||||||||||||||||||||||||||||   |||||||||||||||||||||||||
Sbjct  183734  CCCTAGAAGTAATTTCTAAAAAAAAAAAAAAAAAATCAAGGAAGATGTAATAGTTTTCCA  183675

Query  30775   TAAATTAGAATACCCTACATGTACAATTAAATGAAATGGCTAGTATAGTCTTGAAACCAA  30834
               |||||||||||||||||||| |||||||||||||||||||||||||||||||||||||||
Sbjct  183674  TAAATTAGAATACCCTACATATACAATTAAATGAAATGGCTAGTATAGTCTTGAAACCAA  183615

Query  30835   AACCAGATAAGGTAAATTAAATTCTGTGATATTTTAAAATACTGTAAATTCTGACTAATG  30894
               || |||||||||||||||||||||||||||||||||||||||||||||||||||||||||
Sbjct  183614  AATCAGATAAGGTAAATTAAATTCTGTGATATTTTAAAATACTGTAAATTCTGACTAATG  183555

Query  30895   TGAGTTAATCTCAAAATATGAAATAGTAGATTAACATTGAAAATGCAATAAATAAAATTA  30954
               |||||||||||||| |||||||||||||||||||||||||||||||||||||||||||||
Sbjct  183554  TGAGTTAATCTCAATATATGAAATAGTAGATTAACATTGAAAATGCAATAAATAAAATTA  183495

Query  30955   GCTACCTCAAGAGTTTAATGGaaaaaaaTGTGATTATTGCAATAGATTCAGGAAATTCGT  31014
               |||||||||||||||||||||||||||||||||||||||||||||||||||||||||| |
Sbjct  183494  GCTACCTCAAGAGTTTAATGGAAAAAAATGTGATTATTGCAATAGATTCAGGAAATTCAT  183435

Query  31015   GAATAACATTCACCCTATATTTGTAGGACAACTATCTGATTAA------GTGACCTGTGA  31068
               |||||||||||||||||||||||||||||||||||||||||||      |||||||||||
Sbjct  183434  GAATAACATTCACCCTATATTTGTAGGACAACTATCTGATTAACTTTAAGTGACCTGTGA  183375

Query  31069   CAACCATTTGAATTAATGCTGCTTTCACAGCATATCTCTTGGCTTGTTAAAAACCCGACA  31128
               ||||||||||||||||||||||||||||||||||||||||||||||||||||||||||||
Sbjct  183374  CAACCATTTGAATTAATGCTGCTTTCACAGCATATCTCTTGGCTTGTTAAAAACCCGACA  183315

Query  31129   AGAATTTCCATAACATTAATTTATTTTTAACACCTATATTGGGTGTGAACCCACCATAAA  31188
               ||||||||| ||||||||||||||||||||||||||||||||||||||||||||||||||
Sbjct  183314  AGAATTTCCGTAACATTAATTTATTTTTAACACCTATATTGGGTGTGAACCCACCATAAA  183255

Query  31189   GTTTGCCCACTGAAAAGGTCTGCAATTTGATGCTTTATTAAATTGATACTGTGTGCACCC  31248
               ||||||||||||||||||||| ||||||||||||||||||||||||||||||||||||||
Sbjct  183254  GTTTGCCCACTGAAAAGGTCTACAATTTGATGCTTTATTAAATTGATACTGTGTGCACCC  183195

Query  31249   AACACCACGATCTAATTTAAATATGTTTCCCTCACCCAAGTTCTCTCTTGTGCACTGGCA  31308
               ||||||| |||||||||||||||||||||||||||||||||||||||||| |||||||||
Sbjct  183194  AACACCATGATCTAATTTAAATATGTTTCCCTCACCCAAGTTCTCTCTTGCGCACTGGCA  183135

Query  31309   GTTAATCCCCACTCCCATCTCCAGCCCTAAGCAATACTGCTGTGACATTCCATCTCCATA  31368
               ||||||||||||||||||||||||||||||||||||||||||||||||||||||||||||
Sbjct  183134  GTTAATCCCCACTCCCATCTCCAGCCCTAAGCAATACTGCTGTGACATTCCATCTCCATA  183075

Query  31369   AATTTCCCACTTGCTTTATAGAAATGGACatatatatatat--TTGGAATCTGACTTCCT  31426
               |||||||| ||||||| ||||||||||||||||||||||||  |||||||||||||||||
Sbjct  183074  AATTTCCCGCTTGCTTAATAGAAATGGACATATATATATATATTTGGAATCTGACTTCCT  183015

Query  31427   TCATTTAGCATACTATGTTTGAAGTTAATTGACGTGTTAGCACGTGCTGGTCATGTGTTT  31486
               ||||||||||||||||||||||||||||||||||||||||||||||||||||||||||||
Sbjct  183014  TCATTTAGCATACTATGTTTGAAGTTAATTGACGTGTTAGCACGTGCTGGTCATGTGTTT  182955

Query  31487   TCCTTCATAGTCTGCTGTGTTTATTCATACAGATAGTGTTTATTCATTTATCAGTTAATG  31546
               ||||||||||||||||||||||| ||||||||||||||||||||||||||||||||||||
Sbjct  182954  TCCTTCATAGTCTGCTGTGTTTACTCATACAGATAGTGTTTATTCATTTATCAGTTAATG  182895

Query  31547   GACATTTAATTATTTTGtttttttCTTTGATGAGTAATGTAGCTTTGAGCATTCATATAC  31606
               ||||||||||||||||||| ||||||||||||||||||||||||||||||||||||||||
Sbjct  182894  GACATTTAATTATTTTGTTATTTTCTTTGATGAGTAATGTAGCTTTGAGCATTCATATAC  182835

Query  31607   AGTCATGTAATGCATAATGACATTTTGGTCaaaaaaaattttttttttttCTGAGACCCA  31666
               |||||  |||||||||||||||||||||| ||||||||  ||||||||||||||||||||
Sbjct  182834  AGTCACATAATGCATAATGACATTTTGGT-AAAAAAAA-ATTTTTTTTTTCTGAGACCCA  182777

Query  31667   GGCTGGAGTGCAGTGGCACAATCTCAGCTCACTGGAACCTCCACCTCCCAGGTTTAAGCA  31726
               ||||||||||||||||||||||||| ||||||||||||||||||||||||||||||||||
Sbjct  182776  GGCTGGAGTGCAGTGGCACAATCTCGGCTCACTGGAACCTCCACCTCCCAGGTTTAAGCA  182717

Query  31727   ATTCTCATGCCTCAACCTCCCGAGTAGCTGGGACAACTGGCACACACCACCACGCATGGA  31786
               ||||||||||||||||||||||||||||||||||||||||||||||||||||||| ||||
Sbjct  182716  ATTCTCATGCCTCAACCTCCCGAGTAGCTGGGACAACTGGCACACACCACCACGCCTGGA  182657

Query  31787   TAATTTTTGTATTTTCAGTAGAGACAGGATTTTGCTGTGTTGGTCAGGCTAGTCTCAAAC  31846
               ||||||||||||||||||||||||||||||||||||||||||||||||||||||||||||
Sbjct  182656  TAATTTTTGTATTTTCAGTAGAGACAGGATTTTGCTGTGTTGGTCAGGCTAGTCTCAAAC  182597

Query  31847   TCCTAGCCTCAGGTGATCCGCCCACCTCTGCCTCTCAAAGTGCTGGGATTATAGGCATGA  31906
               ||||||||||||||||||| |||||||||||||| |||||||||||||||||||||||||
Sbjct  182596  TCCTAGCCTCAGGTGATCCACCCACCTCTGCCTCCCAAAGTGCTGGGATTATAGGCATGA  182537

Query  31907   GCCACCACACCCAGCCTAAtttttttAAGAAAC--GAGAACTATTTTCTAAATTACTTTT  31964
               |||||||||||||||||||||||||||||||||  ||||||||||||||||||| |||||
Sbjct  182536  GCCACCACACCCAGCCTAATTTTTTTAAGAAACAAGAGAACTATTTTCTAAATTTCTTTT  182477

Query  31965   GCCAATTTATATTCCTACCATGATGCATAGTACTAATTTCACTGTACAATGTATGGTAGG  32024
               |||||||||||||||||||||||||||||| ||||||||||| |||||||||||||||||
Sbjct  182476  GCCAATTTATATTCCTACCATGATGCATAGCACTAATTTCACCGTACAATGTATGGTAGG  182417

Query  32025   CCCCAATATGTAAGAAATGATGAAAGTAACACATAAAGATTAGTATAAAACAAATGAGAT  32084
               ||||||||||||||||||||||||||||||||||||||||||||||||||||||||||||
Sbjct  182416  CCCCAATATGTAAGAAATGATGAAAGTAACACATAAAGATTAGTATAAAACAAATGAGAT  182357

Query  32085   TATCATTGTTGCTATCATCTTTGTCAAATTCTGAAAACAATCTGAGTATATTTTTATATA  32144
               ||||||||||||||||||||||||||||||||||||||||||||||||||||||||||||
Sbjct  182356  TATCATTGTTGCTATCATCTTTGTCAAATTCTGAAAACAATCTGAGTATATTTTTATATA  182297

Query  32145   AATATGCTTGGCAACATAGCTGAAAAAAGCATTATCAGTTACATTTATCAGTAACAAAGA  32204
               ||||||||||||||||||||||||||||||||||||||||||||||||||||||||||||
Sbjct  182296  AATATGCTTGGCAACATAGCTGAAAAAAGCATTATCAGTTACATTTATCAGTAACAAAGA  182237

Query  32205   CATAAATTTGAAGGGGGAAAAACACTTGTACTAACAACGCAATGTCAGAATTAACATAAA  32264
               ||||||||||||||||||||||||||||||||||||||||||||||||||||||||||||
Sbjct  182236  CATAAATTTGAAGGGGGAAAAACACTTGTACTAACAACGCAATGTCAGAATTAACATAAA  182177

Query  32265   AATTCTGCTGGTCACTTTGGAATATTTAATTGCCTGGGGCAGTGTTTAGTAGACAAATGA  32324
               ||||||||||||||||||||||||||||||||||||||||||||||||||||||||||||
Sbjct  182176  AATTCTGCTGGTCACTTTGGAATATTTAATTGCCTGGGGCAGTGTTTAGTAGACAAATGA  182117

Query  32325   GCAACTATGGAGCACCCAAAGTAGGGGAATCAACAGAACTTGGGTTTCAAAAGTTATCTG  32384
                || ||||||||||||||||||||||||||||||||||||||| ||| ||||||||||||
Sbjct  182116  ACATCTATGGAGCACCCAAAGTAGGGGAATCAACAGAACTTGGATTTGAAAAGTTATCTG  182057

Query  32385   GGTTTAGAGCGTGAAACTTTGTTAGAGGACACACACCTTGCATGAGCGAGGTGACTTGGT  32444
               ||||||||||||||||||||||||||||||||||||||||||||||||||||| ||||||
Sbjct  182056  GGTTTAGAGCGTGAAACTTTGTTAGAGGACACACACCTTGCATGAGCGAGGTGCCTTGGT  181997

Query  32445   GTGTGTGGACGT--------G-TTGGAGGCACAGCATAATGGTGGCTTCCTCCAGAAAGG  32495
               ||||||||||||        | ||||||| ||||||||||||||||||||||||||||||
Sbjct  181996  GTGTGTGGACGTACCATTATGCTTGGAGGTACAGCATAATGGTGGCTTCCTCCAGAAAGG  181937

Query  32496   GACATTTGGGGTGGATTCATTCCATCTAGACAACACAGCCTGATGTGGCATGGACATGAA  32555
               ||||||||||||||||||||||||||||||||||||||||||||||||||||||||||||
Sbjct  181936  GACATTTGGGGTGGATTCATTCCATCTAGACAACACAGCCTGATGTGGCATGGACATGAA  181877

Query  32556   TGGAGGTGAAATGGTCAGTAGTTGAGAGGATCAGTCCTGACAAGGGCCGAAGTGAAAAAC  32615
               |||||||||||||||||||||||||||||||||||||||||||||||||| |||||||||
Sbjct  181876  TGGAGGTGAAATGGTCAGTAGTTGAGAGGATCAGTCCTGACAAGGGCCGAGGTGAAAAAC  181817

Query  32616   CTGGGAACCCCTTCAGGTGCAAAGTCTTCAGTTGAAAAAGGAGGTGGTCACAGGAAATAC  32675
               ||||||||||||||||| |||||| |||||||||||||||||||||||||||||||||||
Sbjct  181816  CTGGGAACCCCTTCAGGAGCAAAGCCTTCAGTTGAAAAAGGAGGTGGTCACAGGAAATAC  181757

Query  32676   TGAGACGGGTCAGCAATGCATGGGAGACAGAGTTCTTGGCCCTGCAGGGTGAGTAGTGTG  32735
               ||||| ||||||||||||||||||||||||||||||||||||||||||||||||||||||
Sbjct  181756  TGAGATGGGTCAGCAATGCATGGGAGACAGAGTTCTTGGCCCTGCAGGGTGAGTAGTGTG  181697

Query  32736   GATTCTCAAGTTTTCTCCTCTCTCCATTAATTTCTTTCCCAATGCAGATGACTTCCATCA  32795
               ||||||||||||||||||||||||||||||||||||||||||||||||||||||||||||
Sbjct  181696  GATTCTCAAGTTTTCTCCTCTCTCCATTAATTTCTTTCCCAATGCAGATGACTTCCATCA  181637

Query  32796   TACAATCTTCAGCAACCTTGAAAGATTGGACAAGCTTCAGCCCACTCTTGAAGGTAAAGG  32855
               |||| |||||||||||||||||||||||||||||||||||||||||||||||||||||||
Sbjct  181636  TACAGTCTTCAGCAACCTTGAAAGATTGGACAAGCTTCAGCCCACTCTTGAAGGTAAAGG  181577

Query  32856   AAGGCAGCTAACAAGACTGGCATCTGGGCTTGGCTGTGCATGTTTGCTATCTTGGAGAAA  32915
               ||||||||||||||||||||||||||||||||||||||| ||||| ||||| ||| ||||
Sbjct  181576  AAGGCAGCTAACAAGACTGGCATCTGGGCTTGGCTGTGCGTGTTTTCTATCATGGGGAAA  181517

Query  32916   TATATATAACACAATATTTATCATTTGAACCTTTTAACCAAAGTGTGCACTCCGTGGCAT  32975
               ||||||||||||| ||||||||||||||||||||||||||||||||||||||||||||||
Sbjct  181516  TATATATAACACATTATTTATCATTTGAACCTTTTAACCAAAGTGTGCACTCCGTGGCAT  181457

Query  32976   TCAATATATTCACAGGGTTGCATAACCAACACCACTATCTACACCCACAATTTTGATGAT  33035
               ||||||||||||||||||||||||||||||||||||||||||||||||||||||||||||
Sbjct  181456  TCAATATATTCACAGGGTTGCATAACCAACACCACTATCTACACCCACAATTTTGATGAT  181397
```

```
Query  33036   TTCTTACAAAACCTTGTCCACAATAAGCAATATAGCGCCTTCCCCCTATTTCCAGCCCAT  33095
               ||||||||||||||||||||||||||||||||||| ||||||||||||||||||||||||
Sbjct  181396  TTCTTACAAAACCTTGTCCACAATAAGCAATATAGTGCCTTCCCCCTATTTCCAGCCCAT  181337

Query  33096   GGTGATTCCTATCC-ACTTTCTCTTGTATGAATTTGACTATTCTAGGCACTTCATGTAAG  33154
               |||||||||||||| |||||||||||||||||||||||||||||||||||||||||||||
Sbjct  181336  GGTGATTCCTATCCCACTTTCTCTTGTATGAATTTGACTATTCTAGGCACTTCATGTAAG  181277

Query  33155   TACAATTATACAATATATTCCTTTTGTGTCTGGCTTATTTCACTAAGCATAATGTTCTCA  33214
               ||| ||||||||||||||||||||||||||||||||||||||||||||||||||||||||
Sbjct  181276  TACGATTATACAATATATTCCTTTTGTGTCTGGCTTATTTCACTAAGCATAATGTTCTCA  181217

Query  33215   ATGTCCACCCATGTTGTATCATCTACCAAAATTATGTTCGTTTTTTACAGATGGATGATG  33274
               ||||||||||||||||||||||||| ||||||||||||||||||||||||||||||||||
Sbjct  181216  ATGTCCACCCATGTTGTATCATCTATCAAAATTATGTTCGTTTTTTACAGATGGATGATG  181157

Query  33275   TAGCATTGCATGTAGACCACCTTGCTTTTATTACATTCATTTGTTCACTGATGGTTGGAT  33334
               |||||||||||| |||||||||||||||||||||||||||||||||||||||||||||||
Sbjct  181156  TAGCATTGCATGCAGACCACCTTGCTTTTATTACATTCATTTGTTCACTGATGGTTGGAT  181097

Query  33335   TATTTCCACCTTTTGCCTCCTGTGAAAAGTGATGCTATAAACATTAGTATACAAGCATCT  33394
               ||||||||||||||| ||||||||||||||||||||| |||||||||||||||| |||||
Sbjct  181096  TATTTCCACCTTTTGGCTCCTGTGAAAAGTGATGCTACAAACATTAGTATACAAACATCT  181037

Query  33395   GTTTGATTCTGTTCTCTATTGTTTGGGGTGCCTAAGAGTAGAGTTCCTGGGTCTAACGG  33454
               |||||||||||||||||||| |||||||||||||||||||||||||||||||| ||| |
Sbjct  181036  GTTTGATTCTGTTCTCTATTCTTTGGGGTGCCTAAGAGTAGAGTTCCTGGGTCCAACAG  180977

Query  33455   GAGTTCTACATTTAACCTTCTGAGCCACTGCAGACTGTTTCTCACAGTGGCTGCAACTTT  33514
               | |||||| ||||||||||||||||||||||||||||||| |||||||||||||||||||
Sbjct  180976  GGGTTCTATATTTAACCTTCTGAGCCACTGCAGACTGTTTTTCACAGTGGCTGCAACTTT  180917

Query  33515   ATCCATTTCTACCATCAATGTATCAGGGTTACAATTTCTTTACATCCTTGTTCACACTTA  33574
               ||||||||||||||||||||||||||||||||||||||||||| ||||| ||||||||||
Sbjct  180916  ATCCATTTCTACCATCAATGTATCAGGGTTACAATTTCTTTACGTCCTTCTTCACACTTA  180857

Query  33575   TTTTCCTTTAAATCATCCTAGTAGGTGTATATTGGTGTCTGCTTGTGTTTTTCATTTGCA  33634
               ||||||||||||||||||||||||||||||||||||| |||||||||||| | |||||||
Sbjct  180856  TTTTCCTTTAAATCATCCTAGTAGGTGTATATTGGTGGCTGCTTGTGTTTATTATTTGCA  180797

Query  33635   TTTCCCTAATGACTAATGATCCTGAGCAGCTTTTCCTGTGCTACTATCTGTGGCTATATC  33694
               ||||||||||||||||||||||||||||||||||||||||||||||||||||||||||||
Sbjct  180796  TTTCCCTAATGACTAATGATCCTGAGCAGCTTTTCCTGTGCTACTATCTGTGGCTATATC  180737

Query  33695   TTCTTTAGGGAAATATATGTTGAAGTCttttgcccatttttaaagagttgtctgattttt  33754
               ||||||||||||||||||||||||||||||||||||||||||||||||||||||||||||
Sbjct  180736  TTCTTTAGGGAAATATATGTTGAAGTCTTTTGCCCATTTTTAAAGAGTTGTCTGATTTTT  180677

Query  33755   atttagttagtttgttgttgtagatttttgAATATATCTTAAATATATTTAAAATATATT  33814
               |||||||||||| |||| |||||||||||||||||||||||||||||||| ||| |||||
Sbjct  180676  ATTTAGTTAGTTGGTTGCTGTAGATTTTTGAATATATCTTAAATATATTTTAAA-ATATT  180618

Query  33815   CTAAATTTTAGTCTCTTACAAGATAAATGATTTGCAAATATTTCCCCCTTTGTGTAGAAC  33874
               ||||||||||||||||||||||||||||||||||||||||||||||||||||||||||||
Sbjct  180617  CTAAATTTTAGTCTCTTACAAGATAAATGATTTGCAAATATTTCCCCCTTTGTGTAGAAC  180558

Query  33875   TTTAGATTCACAAACTTCATTAATTTGTAAGAAATCCTCAGCAGTTGACCCCAAACAGAT  33934
               ||||||||||||||||||||||||||||| ||||||||||||||||||||||||||||||
Sbjct  180557  TTTAGATTCACAAACTTCATTAATTTGTATGAAATCCTCAGCAGTTGACCCCAAACAGAT  180498

Query  33935   AAGACTGAAGCAGTATCTTAGGAATAGTTGAAAGTATGATCACCACAAAACATAAGCGTA  33994
               |||||||||||||||| |||||||||||||||||||||||||||||||||||||||| ||
Sbjct  180497  AAGACTGAAGCAGTATTTTAGGAATAGTTGAAAGTATGATCACCACAAAACATAAGCCTA  180438

Query  33995   ATCAAATCCTGCAAGCTACATGTAAGGCACAATGACAAATAAGGCAGCAAAGGGCCATCT  34054
               ||||||||||||||||||||||||||||||||| ||||||||||||||||||||||||||
Sbjct  180437  ATCAAATCCTGCAAGCTACATGTAAGGCACAATAACAAATAAGGCAGCAAAGGGCCATCT  180378

Query  34055   GGTGATTAGTTCACCACACTTGTTGCAACTGTTTGTGCTGCAGAATTAAAACATACCAGC  34114
               ||||||||||||||||||||||||||||||||||| |||||||| |||||||||||||||
Sbjct  180377  GGTGATTAGTTCACCACACTTGTTGCAACTGTTTGCGCTGCAGAGTTAAAACATACCAGC  180318

Query  34115   ATTCAACCCATGTCTCCTCTCTTGAAGTAAACTGTCGTATGTTGGCTGGCCTGAACAAGC  34174
               ||||||||||||||||||||||||||||||||||| ||||||||||||||||||||||||
Sbjct  180317  ATTCAACCCATGTCTCCTCTCTTGAAGTAAACTGTAGTATGTTGGCTGGCCTGAACAAGC  180258

Query  34175   GTAGATATTCTCCATCCTCAATTAATATGCATGCATGACAAAGAAAAGGAGGCCGGGATG  34234
               |||| ||||||||||||||||||||||||||||||||||||||||||||||||| |||||
Sbjct  180257  GTAGGTATTCTCCATCCTCAATTAATATGCATGCATGACAAAGAAAAGGAGGCCTGGATG  180198

Query  34235   AAAAAATATTGTGTGATTAATAATTATGCTTTAATTAATTTTAAAGGATATAATTTCAGT  34294
               ||||||||||||||||||||||||||||||||||||||||||||||||||||||||||||
Sbjct  180197  AAAAAATATTGTGTGATTAATAATTATGCTTTAATTAATTTTAAAGGATATAATTTCAGT  180138

Query  34295   ACTTCTAATTCTCCCATCAGCAGTTATAACAAAGGATTAGTGAATAAATACCATAGACTG  34354
               ||||||||||||||||||||||||||||||||||||||||||||||||||||||||||||
Sbjct  180137  ACTTCTAATTCTCCCATCAGCAGTTATAACAAAGGATTAGTGAATAAATACCATAGACTG  180078

Query  34355   TTTTGCCTAGAATTGAATCCAATCTGTCTATTAAACTTTGCTTTTATTCAAGTGCAAAAT  34414
               ||||||||||||| ||||||||||||||||||||||||||||||||||||||||||||||
Sbjct  180077  TTTTGCCTAGAATCGAATCCAATCTGTCTATTAAACTTTGCTTTTATTCAAGTGCAAAAT  180018

Query  34415   GCTAAAACACATAATAACTACAGTGACAGCCACTGTGGATCCTCAGAGGCAAAATTAGTC  34474
               ||||||||||||||||||| ||||||||||||||||||||||||||||| |||| |||||
Sbjct  180017  GCTAAAACACATAATAACTGCAGTGACAGCCACTGTGGATCCTCAGAGGTAAAAGTAGTC  179958

Query  34475   TTGGGACATAAATCCTGCAAGCTAATATTGTTTTTACAGGGTTTAGAAAACCATTTAGCT  34534
               ||| ||||||||||||||||||||||||||||||||||||||||||||||||||||||||
Sbjct  179957  TTGCGACATAAATCCTGCAAGCTAATATTGTTTTTACAGGGTTTAGAAAACCATTTAGCT  179898

Query  34535   GGGTTTCAAACCTCACAGTGTGAGCAGTGGGACTCTCATCAAACTATAGCATGTGCTTCA  34594
               ||||||||||||||||||||||||||||||||||||||||||||||||||||||||||||
Sbjct  179897  GGGTTTCAAACCTCACAGTGTGAGCAGTGGGACTCTCATCAAACTATAGCATGTGCTTCA  179838

Query  34595   GTACCATTTGTAGACTGACTCATTCCCATTGCCTTAAGTTGCCATCAGCAAAATGCCAGG  34654
               ||||||||||||||||||||||||||||||  ||||||||||||||||||||||||||||
Sbjct  179837  GTACCATTTGTAGACTGACTCATTCCCATTTTCTTAAGTTGCCATCAGCAAAATGCCAGG  179778

Query  34655   GACTCTATTTCTTGCTCCTTAGCTCCTCGTTCTTGCTTGTCTTTCCACGAGGGAAGATTT  34714
               |||||||||||||||||||||||||||||||||||| |||||||||||||||||||||||
Sbjct  179777  GACTCTATTTCTTGCTCCTTAGCTCCTCGTTCTTGCCTGTCTTTCCACGAGGGAAGATTT  179718

Query  34715   TCTAGCAGGAGCTCAAGCTGTGCTTTTAATGAAACACATCCACACACACTGTCCTGTTGT  34774
               |||||||||||||||| |||||||||||||||||||||||||||||||||||||||||||
Sbjct  179717  TCTAGCAGGAGCTCAAACTGTGCTTTTAATGAAACACATCCACACACACTGTCCTGTTGT  179658

Query  34775   CCACATTAAGCAGAGCTCCCTGAATAACTCATGAACAAAAGCATCTATGACTAACTGTTG  34834
               ||||||||||||||||||||||||||||||||||||||||||||||||||||||||||||
Sbjct  179657  CCACATTAAGCAGAGCTCCCTGAATAACTCATGAACAAAAGCATCTATGACTAACTGTTG  179598

Query  34835   CTGTGTGTCCTCCTAGCCTCTGAGGAGTCTCTAGTTCACAAGGACAGAGGAGATGGAGAG  34894
               || |||||||||||||||||||||||||||||||||||||||||||||||||||||||||
Sbjct  179597  CTCTGTGTCCTCCTAGCCTCTGAGGAGTCTCTAGTTCACAAGGACAGAGGAGATGGAGAG  179538

Query  34895   AGGCCAGTCAACGTGAGGGTAAGGTTGCCTTGCTTTCTCTGAAATAGAAATGTTCCTTTC  34954
               ||||||||||||| ||||||||||||||||||||||||||||||||||||||||||||||
Sbjct  179537  AGGCCAGTCAACGCGAGGGTAAGGTTGCCTTGCTTTCTCTGAAATAGAAATGTTCCTTTC  179478

Query  34955   TTGGTGTCTTTCTTTTTCAACTGACTTTACATGTGAAAAAATGACAATGTCCATGACAGG  35014
               ||||||||||||||||||||||||||||||||||||||||||||||||||||||||||||
Sbjct  179477  TTGGTGTCTTTCTTTTTCAACTGACTTTACATGTGAAAAAATGACAATGTCCATGACAGG  179418

Query  35015   TATTAAATGCAGTTTTCTGAGGGGGAGGAAGAAGTGACTCTTAGCAACTGATATGTAATC  35074
               ||||||||||||||||||||||||||||||||||||||||||||||||||||||||||||
Sbjct  179417  TATTAAATGCAGTTTTCTGAGGGGGAGGAAGAAGTGACTCTTAGCAACTGATATGTAATC  179358

Query  35075   CAAAATGGCATTTAGCTATGACGGCTTCAGGTTGTAGACTGTATCCTTGGGATCCTTGTC  35134
               ||||||||||||||||||||||||||||||||||||||||||||||||||| ||||||||
Sbjct  179357  CAAAATGGCATTTAGCTATGACGGCTTCAGGTTGTAGACTGTATCCTTGGGGTCCTTGTC  179298

Query  35135   CTTGGAAGCAATGTCTTCTCCTTGGATTCAGTATTTTGCACTTGCCAACCTACGTGGACC  35194
               ||||||||||||||||||||||||||||||||||||||||||||||||||||||||||||
Sbjct  179297  CTTGGAAGCAATGTCTTCTCCTTGGATTCAGTATTTTGCACTTGCCAACCTACGTGGACC  179238

Query  35195   TGAGAGATACACCATCCAGAAGCTGATGTCTTTTCCAGTGTGTATCCTACCCTTGTCTTG  35254
               |||||||||| ||||||||||||||||||||||||||||||||||||||||||||| |||
Sbjct  179237  TGAGAGATACGCCATCCAGAAGCTGATGTCTTTTCCAGTGTGTATCCTACCCTTGTTTTG  179178

Query  35255   GAGGCCTTGAAGTTGACTACACTTTCTGATCAAGTTTTCAGTATTCATTGAGAGAAACAG  35314
               |||||||||||||||||||||||||||||||||||||||| |||||||||||||||||||
Sbjct  179177  GAGGCCTTGAAGTTGACTACACTTTCTGATCAAGTTTTCAATATTCATTGAGAGAAACAG  179118

Query  35315   AGCCTTGTGCAAACAATCCACAACATGACATACCCCTCAAAAAGTTTGTTTCTGTATTG  35374
               |||||||||||||||||||||||||||||||||||||||||||| |||||||||||||||
Sbjct  179117  AGCCTTGTGCAAACAATCCACAACATGACATACCCCTCAAAAAGCTTTGTTTCTGTATTG  179058

Query  35375   CAGGTGGTGCAGGTGGCCCCTCTGAGGCGTGAATCTAGTAAGTATTCTGGAATCACTTGC  35434
               ||||||||||||||||||||||||||||||||||||||||||||||||||||||||||||
Sbjct  179057  CAGGTGGTGCAGGTGGCCCCTCTGAGGCGTGAATCTAGTAAGTATTCTGGAATCACTTGC  178998

Query  35435   CAAGAAAACAATCTGGATGCCAAGAAAGGTGTGGCATCCTTGCCTGGTTTCAATGTGAAG  35494
               ||||||||||||||||||||||||||||||||||||||||||||||||||||||||||||
Sbjct  178997  CAAGAAAACAATCTGGATGCCAAGAAAGGTGTGGCATCCTTGCCTGGTTTCAATGTGAAG  178938

Query  35495   AGCCACCCTGATCCTGGGATTGTGATAGGAATAAGTATAGGGGAAGTGtttttttAAAAC  35554
               |||| |||||||||||||||||||||||||||||||||||||||||||||||||||||||
Sbjct  178937  AGCCTCCCTGATCCTGGGATTGTGATAGGAATAAGTATAGGGGAAGTGTTTTTTTAAAAC  178878

Query  35555   CTGAATTCCCCAGGGAAAAATTATGGCCAAATTTTGAGGAAGCAGCTGTGCTCCCTTTTG  35614
               ||||||||||||||||||||||||||||||||||||||||||||||||||||||||||||
Sbjct  178877  CTGAATTCCCCAGGGAAAAATTATGGCCAAATTTTGAGGAAGCAGCTGTGCTCCCTTTTG  178818
```

```
Query  35615   GGTGGTGCTGAGTTGGGTGCTTGAGGATTGGTGGTGTCTTGTTTGAGGCTGCATCgtgtg  35674
               |||||||| ||||||||||||||||||||||||||||||||| |||||||||||||||||
Sbjct  178817  GGTGGTGCCGAGTTGGGTGCTTGAGGATTGGTGGTGTCTTGTGTGAGGCTGCATCGTGTG  178758

Query  35675   gtgtgaatgtgtgtgtttctgtacaggtgaggctgtgtgtTTTCTCAGGAGAGATTTCCC  35734
               ||||||||||||||||||||||||||||||||||||||||||||||||||||||||||||
Sbjct  178757  GTGTGAATGTGTGTGTTTCTGTACAGGTGAGGCTGTGTGTTTTCTCAGGAGAGATTTCCC  178698

Query  35735   ACTTATACAACCCAATCACCAGTGTCCACTTCTAACAATAAAATCCACCCCCGCTCTACT  35794
               |||||||||||||||||||||||||||||||||||||||||||||||||||| |||||||
Sbjct  178697  ACTTATACAACCCAATCACCAGTGTCCACTTCTAACAATAAAATCCACCCCCACTCTACT  178638

Query  35795   CTCTCTGTACAGTGACTCCTCTACCCTCACCAGAGCCATCCCCGGGTCTGCCTTATTATC  35854
                |||||||||||||||||||| ||||||||||||||||||||| ||||||||||||||||
Sbjct  178637  GTCTCTGTACAGTGACTCCTCCACCCTCACCAGAGCCATCCCCAGGTCTGCCTTATTATC  178578

Query  35855   CCGACTACTCAGGTGGAGAACCTGAAGGGCCAAGGGAGTGGCCCCAGCTCCTGAGTTCCT  35914
               || ||| |||||||||||||||||||||||||||||||||||||||||||| || |||||
Sbjct  178577  CCCACTGCTCAGGTGGAGAACCTGAAGGGCCAAGGGAGTGGCCCCAGCTCCCGACTTCCT  178518

Query  35915   GAATGAAAAAGTGAAAACACGAACCCAGGAGTGTGGGCCAGTGCTGACACTGACATGCAC  35974
               |||||||||||||||||||||||||||||||||||||||||||||||| |||||||||||
Sbjct  178517  GAATGAAAAAGTGAAAACACGAACCCAGGAGTGTGGGCCAGTGCTGACGCTGACATGCAC  178458

Query  35975   TTAGTCATGGAGTGTTCACCACCACACAGGGAGTTCAGCATTCATCTATAAGCCCTAAAG  36034
               |||||||||| ||||||||||||||||||||||| |||||||||| ||||||||||||||
Sbjct  178457  TTAGTCATGGGGTGTTCACCACCACACAGGGAGTCCAGCATTCATGTATAAGCCCTAAAG  178398

Query  36035   CACCGAGCCCAAAAGGCCCCAGACACTGCCCATCATCATAAAGTGGCCTCTGTGGTCACA  36094
               |||||||||||||||||||||||||||||||||||||||||||||||||| |||||||||
Sbjct  178397  CACCGAGCCCAAAAGGCCCCAGACACTGCCCATCATCATAAAGTGGCCTCCGTGGTCACA  178338

Query  36095   CAACCCAGGGCAGTTATAGGCTCATCTCCCCACGGACAGGCATAGTCATCAGTGTGTCAA  36154
               ||||||||||||||||||||||||||||||||||||| ||||||||||||||||||||||
Sbjct  178337  CAACCCAGGGCAGTTATAGGCTCATCTCCCCACGGACCGGCATAGTCATCAGTGTGTCAA  178278

Query  36155   AAGCACAAAGATCCCCAGGTGTTTTGCTCAGCTCACAGATC----ttttttttttAACTT  36210
               |||||||||||||||||||||||| ||||||||||||||||    |||||||||||||||
Sbjct  178277  AAGCACAAAGATCCCCAGGTGTTTGGCTCAGCTCACAGATCCCCCTTTTTTTTTTAACTT  178218

Query  36211   TTAAGTTCAGGGGTACATGTGCAGGATGTGCAGGTTTGCTACATATAGAAATGTGTGTCA  36270
               |||||||||||||||||||||||||||||||||||||||||||||||  ||||||||| |
Sbjct  178217  TTAAGTTCAGGGGTACATGTGCAGGATGTGCAGGTTTGCTACATATATGAATGTGTGTTA  178158

Query  36271   TGAGAGTTTGTTGTACAGATTATTGCATCACCCATACATTGAGCCTAATATCAGCTATTT  36330
               |||||||||||||||||||||||||||||||||||| |||||| |||||||||| |||||
Sbjct  178157  TGAGAGTTTGTTGTACAGATTATTGCATCACCCATATATTGAGGCTAATATCAGTTATTT  178098

Query  36331   TTCCTGATCCTCCCCCTCCTCCCACCTCCCACCCTCC---AGTGGGCCCCACGCTCACAA  36387
               |||||||||| ||||||||||||||||||||||||||   ||| ||||||||||||||||
Sbjct  178097  TTCCTGATCCACCCCCTCCTCCCACCTCCCACCCTCCTCCAGTAGGCCCCACGCTCACAA  178038

Query  36388   ATTCTAAGAGGAGTGGGGGACCACAAATGCCAGTGTGGCCCACTTCAGTTGTGAAGTTAA  36447
               ||||||||||||||||||||||||||| ||||||||||||||||||||||||||||||||
Sbjct  178037  ATTCTAAGAGGAGTGGGGGACCACAAAGGCCAGTGTGGCCCACTTCAGTTGTGAAGTTAA  177978

Query  36448   TTTGCTCAGCAGCTGGCCAAAGCCTGTAAGGATGGGTGATGTATTTTAGTAGATTTAGTA  36507
               | ||||||||| |||||||||| || ||||||||||||||||||||||||||||||||||
Sbjct  177977  TCTGCTCAGCAACTGGCCAAAGTCTATAAGGATGGGTGATGTATTTTAGTAGATTTAGTA  177918

Query  36508   ATACTATCTTCCCAAGCCCTAAAATGCTCAAAACCTGCCAGCCAAAAATGGTGAGGAGGG  36567
               |||||||||||||||||||||||||||||||| |||||||||||||||||||||||||||
Sbjct  177917  ATACTATCTTCCCAAGCCCTAAAATGCTCAAATCCTGCCAGCCAAAAATGGTGAGGAGGG  177858

Query  36568   ACAGATAGGAACTCTGTGTGGCACTTGGTTATTAGCCTGGCTTCCATCCCTTAGTGGCAA  36627
               ||||||||||||||||||||||||||||||||||||||||||||||||||||||||||||
Sbjct  177857  ACAGATAGGAACTCTGTGTGGCACTTGGTTATTAGCCTGGCTTCCATCCCTTAGTGGCAA  177798

Query  36628   CTCTCTTGTATATGTGGGTTAAAGACCCTCAGCCTCAAGCCAAGCCTCCTCCATGAGGAG  36687
               ||||||||||||||||||||||||||||||||||||||||||||||||||||||||||||
Sbjct  177797  CTCTCTTGTATATGTGGGTTAAAGACCCTCAGCCTCAAGCCAAGCCTCCTCCATGAGGAG  177738

Query  36688   CCATCTCACTATTGACTGGCTAGTGCCGGGTATGGCCACCAGCCCAACTGAAACAAAATG  36747
               ||||||||||||||||||||||||||||||||||||||||||||||||||||||||||||
Sbjct  177737  CCATCTCACTATTGACTGGCTAGTGCCGGGTATGGCCACCAGCCCAACTGAAACAAAATG  177678

Query  36748   TTGCTTTAAAACAAGTGTAAATCTCATACA----ACAGGCAAATGCAGAAGCAATGTGGT  36803
               ||||||||||||||||||||||||||||||    ||||||||||||||||||| ||||||
Sbjct  177677  TTGCTTTAAAACAAGTGTAAATCTCATACATACAACAGGCAAATGCAGAAGCAGTGTGGT  177618

Query  36804   CTCGCAAGTTGTAAAGAGGACAGTCGCAATTTTGCTGGACTTCAACCTGGGTAGAAGACA  36863
               ||||||||||||||||||||||||||||||||||||||||||||||||||||||||||||
Sbjct  177617  CTCGCAAGTTGTAAAGAGGACAGTCGCAATTTTGCTGGACTTCAACCTGGGTAGAAGACA  177558

Query  36864   TGAGGGAACTCTGTCACTGAATCACGGCAGAGTTCAAGGCCACTTGTAGACTATTTCATG  36923
               |||||||||||||||||||||||||||||||||||||||||||||| |||||||||||||
Sbjct  177557  TGAGGGAACTCTGTCACTGAATCACGGCAGAGTTCAAGGCCACTTGCAGACTATTTCATG  177498

Query  36924   TTACAGAAGGTAGCCTTTAGCTACTAAGCAAAGGCCTCGGTTTCTCATTTCTTTCCTGTT  36983
               ||||||||||| |||||||||||||||||||||||||| |||||||||||||||||||||
Sbjct  177497  TTACAGAAGGTGGCCTTTAGCTACTAAGCAAAGGCCTCTGTTTCTCATTTCTTTCCTGTT  177438

Query  36984   CATCTTCTTGGTCATCCTTCTTCCGCAAGGGAAACGAGCCCAAGCAAAAGGCAGTTTCAA  37043
               ||||||||||||||||||||||||||||||||||||||||||||||||||||||||||||
Sbjct  177437  CATCTTCTTGGTCATCCTTCTTCCGCAAGGGAAACGAGCCCAAGCAAAAGGCAGTTTCAA  177378

Query  37044   TATTAATTTGACCGAGGTTTTGTGCAGTTTATTATCATCCAGGTAATCAGGTGCAACCCA  37103
               ||||||||||||||||||||||||||||||||||||||||||||||||||||||||| | 
Sbjct  177377  TATTAATTTGACCGAGGTTTTGTGCAGTTTATTATCATCCAGGTAATCAGGTGCAACTCG  177318

Query  37104   GTCTGCCTAGCAGCCCCCTCTCTCTGCTCTGTGTTTTCATTTAATAAACATTTTGGTCT  37163
               ||||||||||||||||||| ||||||||||||||||||||||||||||||||||||||||
Sbjct  177317  GTCTGCCTAGCAGCCCCCTATCTCTGCTCTGTGTTTTCATTTAATAAACATTTTGGTCT  177258

Query  37164   ACTTACTATGTGCTAGATTTTCTCGAGACCAAGTAAATGAGATAGAATCTTTATGTTGGC  37223
               ||||||||||||||||||||||||||||||||||||||||||||||||||||||||||||
Sbjct  177257  ACTTACTATGTGCTAGATTTTCTCGAGACCAAGTAAATGAGATAGAATCTTTATGTTGGC  177198

Query  37224   AGCTAAGTTAGATTTAACATAACTGACAAAAATTAAAATTTCTGATTTCTTGTAAAAAT-  37282
               |||||||||||||||||||||||||||||||||||||||||||||||||||||||||||
Sbjct  177197  AGCTAAGTTAGATTTAACATAACTGACAAAAATTAAAATTTCTGATTTCTTGTAAAAATA  177138

Query  37283   ---TGTATGTGTGAATGCATACTGAAAGTAAAGTGGTTAACaaaaaaaaTCACACTTGCA  37339
                  ||||||||||||||||||||||| ||||||||| ||| ||||    |||||||||||
Sbjct  177137  TTTGTATGTGTGAATGCATACTGAATGTAAAGTGGATAA-AAAAC---TCACACTTGCA  177082

Query  37340   CTCATGGAAGGCTTTTCATGAATTTGTCAATTTCTAtttttttATATTTCCCCACTTCAC  37399
               ||||||||||||||||||||||||||||||||| |||||||| |||||||||||||||||
Sbjct  177081  CTCATGGAAGGCTTTTCATGAATTTGTCAATTTTTATTTTTT-ATATTTCCCCACTTCAC  177023

Query  37400   TGGATAATGCATATCTGAACCTGGAAACTGATTCCCACTGTAGAAAGTGTTCTGAGCCAC  37459
                |||||||||||| |||||||||||||||||||||||||| |||||||||||||||||||
Sbjct  177022  CGGATAATGCATACCTGAACCTGGAAACTGATTCCCACTGCAGAAAGTGTTCTGAGCCAC  176963

Query  37460   ATCCCTTAGCTTCACTAGTGCAGGTCCACCTGGGAGTATGTCCCAGCATCAGCTTGGCCC  37519
               |||||||||||||||||||||||||||||||||||| |||||||||||||||||||||||
Sbjct  176962  ATCCCTTAGCTTCACTAGTGCAGGTCCACCTGGGAGGATGTCCCAGCATCAGCTTGGCCC  176903

Query  37520   ATGCTGTGATAAGCCACCTCCATGCACCACACCAAGCAAGCCCCTGGGTGATTCACAGTC  37579
               |||||||||| |||||||||||||||||||||||||||||||||||||||||||||||||
Sbjct  176902  ATGCTGTGATCAGCCACCTCCATGCACCACACCAAGCAAGCCCCTGGGTGATTCACAGTC  176843

Query  37580   TCCACCACCAGGGCACTGACCTTTATTCTGTGTTCTTCAAGCTCCCCATGGGGACACCAT  37639
               ||||| ||||||||||||||||| | |||||||||||| ||||||||||| ||||||| |
Sbjct  176842  TCCACTACCAGGGCACTGACCTTAACTCTGTGTTCTTCTAGCTCCCCATGAGGACACCGT  176783

Query  37640   CCACGACATCACGAACGAGGACGCCGTC-CACGACACCACCGACGAGGACGCCGTCCATG  37698
                ||| ||||||| |||||||||||| || ||||| | |||  ||||||||||| | || |
Sbjct  176782  ACACAACATCACTAACGAGGACGCC-TCACACGATATCACTAACGAGGACGCCATACACG  176724

Query  37699   GCACCGCCGACGAGGACGCCGTCCAGGGCATCGCCGACGAGGACGCCGTCCACGGCATCG  37758
                |  |||  |||||||||| ||||| ||||||||  | |||| | ||| | | |||||||
Sbjct  176723  ACGTCGCTAACGAGGACGCTGTCCACGGCATCGCTAATGAGGCCACCGACAAGGGCATCG  176664

Query  37759   CTGACTGGGACGCCATCCAGGGCCTCGCTGACAGGGACGCCGTCCACGGCTTCGCTGATG  37818
               |  ||  |||| ||  ||||||| ||||  ||  || ||||||||||||| ||||  | |
Sbjct  176663  CCAACGAGGACACCGCCCAGGGCATCGCCAACGTGGTCGCCGTCCACGGCATCGCCAACG  176604

Query  37819   GGGACGCCGTCAAGGGCATCGCTGACGGGGACGCCACCCAGGGCATCGCTGAAGGGGACG  37878
                ||| |||| | ||||||||||  ||| ||| |||  ||| ||||| ||| | | ||| |
Sbjct  176603  AGGATGCCGCCCAGGGCATCGCCAACGAGGATGCCGTCCACGGCATTGCTAACGAGGATG  176544

Query  37879   ACGTCCATGGCATCGCTGATG  37899
                ||| || |||||| || |||
Sbjct  176543  CCGTGCACGGCATCACTAATG  176523
```

Figure S3. Nucleotide sequence alignment of RNA gene FAM230C with a segment of ch22 from the chimpanzee genome. The alignment begins at FAM230C, position 20796, which represents the 3rd TBTA repeat of FAM230C (as shown by RepeatMasker analysis, Table 3 ref. 12), and ends 29 nt from the 3'end of FAM230C, 37928 nt. Alignment by NCBI Blast Basic Local Alignment Search Tool (https://blast.ncbi.nlm.nih.gov/Blast.cgi?CMD=Web&PAGE_TYPE=BlastHome).
