# Supplementary material for: Formation of a Family of Long Intergenic Noncoding RNA Genes with an Embedded Translocation Breakpoint Motif in Human Chromosomal Low Copy Repeats of 22q11.2—Some Surprises and Questions
Source: Noncoding RNA. 2018 Jul 20;4(3):16. doi: 10.3390/ncrna4030016 (PMC6162681; doi:10.3390/ncrna4030016)
Supplement: Supplementary file 1 [file ncrna-04-00016-s001.zip › Table S1.pdf]

Formation of a family of long intergenic non-coding RNA genes with an embedded translocation breakpoint motif in human chromosomal Low Copy Repeats of 22q11.2 - some surprises and questions

Nicholas Delihas
Department of Molecular Genetics and Microbiology
School of Medicine
Stony Brook University
Stony Brook, New York, 11794-5222 USA

Supplementary File 1 (reproduced from reference 13)

Table S1. Properties of lincRNA genes in chr22*

lincRNA gene, chromosomal location, length; LCR22 position	lincRNA nt positions	FAM230C nt positions
1. LINC01663 (AC008103.3)	314 -21974	17003- 37928
chr22: 18,872,943-18,895,007		
22065 bp; LCR22A		
2. LINC01660 (AC011718.2)	4028-21304	16903-37928
chr22: 18,361,223-18,391,705		
30,483 bp; LCR22A		
3. LINC01662 (AC008132.15)	1-16346	17872-37928
Chr22: 18,733,314-18,758,506		
25,913 bp LCR22A		
4. FAM230B	1-16529	17410-37928
chr:22: 21,167,158-21,192,756		
25,599 bp; LCR22D		
5. AP000552.1 (KB-1183D5.13)	1-16588	17408- 37928
chr22: 21,300,390-21,325,642		
25,253 bp; LCR22D		
6. AC007731.1	1- 16551	17401-37928
chr22:20,338,205-20,354,972		
16,768 bp; LCR22B		
7. AC008079.1	380- 16994	16994-37928
chr: 22: 18,177,438-18,206,515		
29,078 bp; LCR22A		
8. LINC01658 (AP000345.1)	1- 16761	16668-37928
chr22: 23461486-23487580		
26,095 bp; LCR22F		

* Most genes have multiple transcripts. The Transcript Support Levels (TSL) (from Ensembl) for each gene vary from 1-5, depending on the transcript.
